# Is a Persistent Global Bias Necessary for the Establishment of Planar Cell Polarity?

**DOI:** 10.1371/journal.pone.0060064

**Published:** 2013-04-08

**Authors:** Sabine Fischer, Paul Houston, Nicholas A. M. Monk, Markus R. Owen

**Affiliations:** Centre for Mathematical Medicine and Biology, School of Mathematical Sciences, University of Nottingham, Nottingham, United Kingdom; Centre for Genomic Regulation (CRG), Universitat Pompeu Fabra, Spain

## Abstract

Planar cell polarity (PCP)–the coordinated polarisation of a whole field of cells within the plane of a tissue–relies on the interaction of three modules: a global module that couples individual cellular polarity to the tissue axis, a local module that aligns the axis of polarisation of neighbouring cells, and a readout module that directs the correct outgrowth of PCP-regulated structures such as hairs and bristles. While much is known about the molecular components that are required for PCP, the functional details of–and interactions between–the modules remain unclear. In this work, we perform a mathematical and computational analysis of two previously proposed computational models of the local module (Amonlirdviman et al., Science, 307, 2005; Le Garrec et al., Dev. Dyn., 235, 2006). Both models can reproduce wild-type and mutant phenotypes of PCP observed in the *Drosophila* wing under the assumption that a tissue-wide polarity cue from the global module persists throughout the development of PCP. We demonstrate that both models can also generate tissue-level PCP when provided with only a transient initial polarity cue. However, in these models such transient cues are not sufficient to ensure robustness of the resulting cellular polarisation.

## Introduction

During embryonic development, the correct formation of tissues and organs requires coordinated rearrangements of cells, which rely on the polarisation of the cells along their apical-basal axis and in many epithelia also within the plane of the tissue. The latter is commonly referred to as planar cell polarity (PCP). Disruption of PCP significantly affects morphogenetic events such as gastrulation and neurulation [1] and impairs body functions such as polarised ciliary beating [2], leading to a variety of diseases including congenital deafness syndromes, neural tube closure defects, respiratory diseases and polycystic kidneys [3].

The fruit fly *Drosophila melanogaster* is an important model organism for studying the mechanism of PCP establishment, since it displays overt PCP features on all adult external structures. The most obvious examples of this are the orientation of the ommatidia in the eyes and the alignment of hairs on the wings and the abdomen. In all of these tissues, PCP is believed to be controlled by interactions between three modules [4], [5]. A global module provides a tissue-wide directional cue that links cellular polarity to the tissue axis; a local module enables cells to align their polarity with their neighbours; and the third module performs the readout. Although the existence of these modules is commonly accepted, the molecular details of the global module are controversial and the interactions of the three modules remain unclear. Initially it was assumed that the three modules worked in a linear sequence: the global module would affect only the local module which would in turn provide the information for the readout. However, recent results point increasingly towards more complex network type interactions in which both the global and the local module directly affect the readout as well as each other.

**Figure 1:**
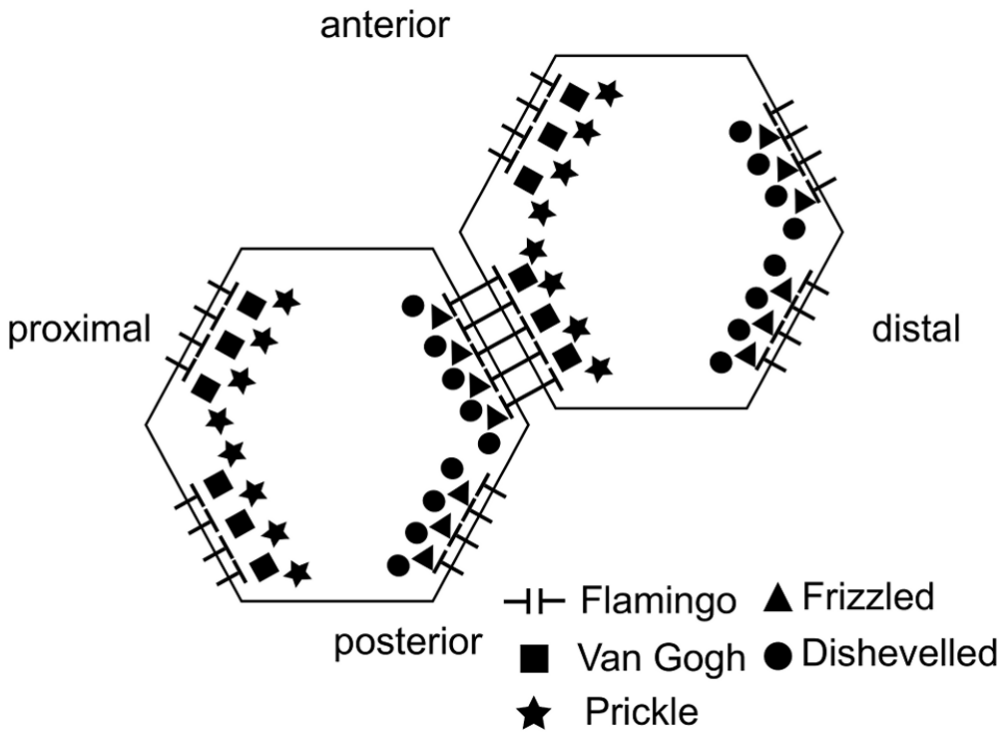
Localisation of the core planar cell polarity proteins at the cell edges.

To date, much emphasis has been placed on the local module, and a range of experimental evidence has revealed that a system of interacting proteins centred around the transmembrane protein Frizzled (Fz) plays a key role. This group of proteins – often referred to as core proteins – includes the atypical cadherin Flamingo (Fmi), the transmembrane protein Van Gogh (Vang; also known as Strabismus) and the cytoplasmic proteins Dishevelled (Dsh) and Prickle (Pk). During the establishment of PCP these five proteins acquire an asymmetric distribution within cells. In the *Drosophila* pupal wing, shortly before hair formation, Fz and Dsh become localised to the distal membrane of each cell, while Vang and Pk colocalise in the proximal membranes. Fmi occurs in both the proximal and the distal membrane, but not anterior or posterior [6]. Figure 1 shows an illustration of the protein distributions.

While the identities of the key molecular species involved in the local module are well established, the way in which they interact to establish their overt patterns of asymmetric localisation is less clear. In recent years two computational models for the local module in the *Drosophila* wing have been proposed. These models incorporate distinct subsets of the core proteins and explore different proposals for their interactions [7], [8]. A common feature of the two models is that the interactions between the core proteins are biased within each cell by a tissue-wide polarity cue from the global module. Importantly, this cue persists throughout the whole process of local cell polarisation, and is “read” and amplified by a feedback loop which consists of interactions between the core proteins. The two models differ in the type of persistent global bias and the details of their feedback mechanisms. Both models aim to reproduce the wild type asymmetric distribution of the core proteins, as shown in Figure 1, as well as the patterns around mosaic cell clones which lack or over-express one of the core proteins.

These models are of great interest since they can produce patterns of PCP that mimic those observed experimentally. Due to the complexity of the interactions it was however not evident how polarity is coordinated across the tissue in these models. Therefore, an important question arises: what are the relative roles played by the persistent global polarity cue and by the local feedback amplification mechanism? An analysis of the models with respect to the wild type polarity will give insight into the relative importance of the persistent global bias and the feedback loop for the establishment of PCP, while focusing on the mutant conditions would address the differences between the feedback loops. In this work we are interested in the interplay of the global and the local module and therefore we consider the wild type situation. Our aim is to reveal the mechanisms that are at the core of these rather complex models. In the following we will introduce the two models in detail and analyse their capability of reproducing the wild type pattern of PCP in one and two spatial dimensions. We find that, despite the differences in molecular details, the basic mechanisms in these models are the same. In both models, robust long-range coordination of polarity relies on the persistent global bias and the feedback mechanisms enhance the strength of polarity. To generate polarity, a small initial imbalance in the cells is sufficient; to ensure robustness the global bias is required. Our results lead to the conclusion that both feedback mechanisms introduce bistable switches across membranes but no feedback within the cells.

## Models

In this work, we analyse the mechanisms proposed by Amonlirdviman *et al.* [7], [9] and Le Garrec *et al.* [8] (applied to the *Drosophila* eye in [10]). Both have a common general structure consisting of a persistent imposed global bias which is amplified by a feedback mechanism, that is based on protein complex formation. To globally bias the polarity of the cells Amonlirdviman *et al.* consider two different mechanisms, a cell intrinsic polarity in the dissociation rates for certain complexes and a polarity for the diffusion of certain proteins and complexes [7]. They find that both versions of their model give similar results. In Le Garrec *et al.* the global module is introduced by imposing a ligand gradient over the whole tissue [8]. For the feedback mechanism the two approaches include different members of the core proteins and assume different interactions as described below.

### Model A

**Figure 2:**
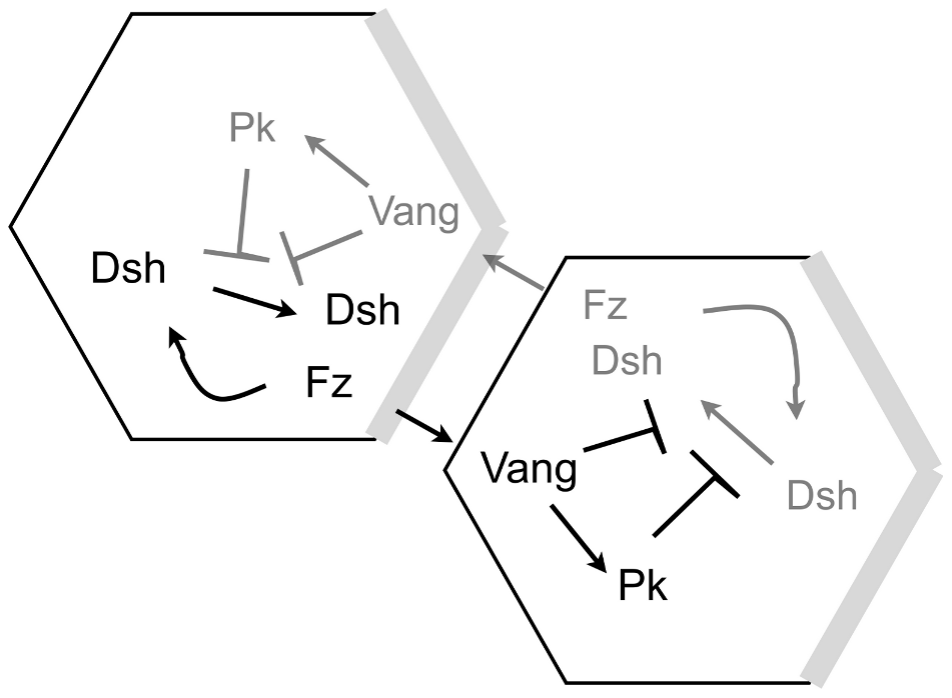
Feedback loop and global bias of Model A. The amounts of proteins in black are higher than the amounts of proteins in grey. Arrows represent recruitment of proteins, T-signs inhibition. The grey regions at the distal sides of the cell indicate where the persistent global bias affects the dissociation rate of Dsh. This figure was reproduced from Fig 2B in [7].

This model is based on the mechanism proposed by Amonlirdviman *et al.* [7], which includes the protein interactions illustrated in Figure 2.

Assuming that the proteins colocalise by forming complexes, the model can be summarised by the following reactions.
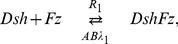
(1)

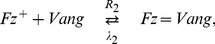
(2)

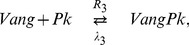
(3)

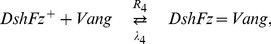
(4)

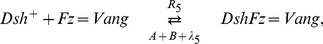
(5)

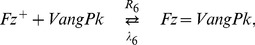
(6)

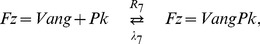
(7)

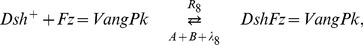
(8)

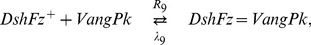
(9)

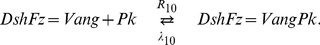
(10)


For each equation 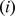 with 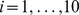, there is a forward reaction rate 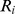 and a backward reaction rate 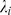. The superscript 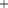 emphasises that the two reactants are in different cells, binding over the cell membrane to form a cell bridging complex which is indicated by = . We adopt the notation that the cell bridging complexes belong to the same cell as their Vang part. The different proteins and complexes have different regions in which they can move. Dsh and Pk can be found in the cytoplasm. Fz, Dsh, DshFz and VangPk can move along the whole membrane of a cell, while the cell bridging complexes are restricted to the part of the membrane that is common to the two cells they connect.

Out of the two mechanisms Amonlirdviman *et al.* proposed for their persistent global bias, we have implemented the bias in dissociation rates. In this scheme, the rates of dissociation of Dsh from Dsh-containing complexes in a region of the distal side of each cell are decreased by multiplying the backward reaction rates of equations (1),(5) and (8) by a factor 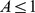 with



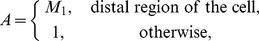
and 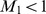. In [9] the persistent global bias was refined from a step function to an intracellular gradient, allowing different directions of the bias in clones that are assumed to interfere with the global module. However, for the purpose of this paper it is sufficient to consider the stepwise global bias along the proximal-distal axis of a cell.

The amplification of this imposed polarity by the local module is achieved in this model by a feedback loop, that consists of Vang and its complexes inhibiting the recruitment of Dsh to complexes. As shown in Figure 2 this inhibition occurs within the same cell. This implies that if we have Vang or its complexes in a given cell, the recruitment of Dsh in that same cell is inhibited, not the recruitment of Dsh from the neighbouring cell to this Vang-complex.

In the equations the feedback is represented by an increase of the backward reaction rates of all the reactions in which Dsh binds to Fz or Fz complexes, namely reactions (1), (5) and (8). To this end, those backward reaction rates are multiplied by a factor 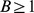 with
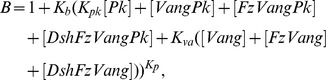
where 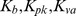 and 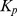 are positive constants. We see that 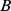 is an increasing function of the concentrations of Pk, Vang and their complexes in the same cell as Dsh.

We are interested specifically in the relative importance of the persistent global bias and the feedback loop in this model for the establishment of PCP. To this end, we analyse two discretisations of the model and conduct simulations of the full spatial system. In our one-dimensional discretisation we assume each cell has two sides, left and right, with certain amounts of the protein and protein complexes, a representation we have previously applied in [11]. In this setting, diffusion is implemented as exchange between the two sides of the cell. This approach enables us to determine which parameter combinations yield polarity, and which yield a homogeneous unpolarised steady state in the one-dimensional model. The two-dimensional discretisation assumes that each cell is hexagonal and consists of six compartments and that intracellular diffusion occurs between neighbouring compartments. This two-dimensional version of the model introduces the possibility of different types of polarisation, towards either a side or a vertex of a cell (see Results section). In Section A.3 of Supporting Information S1 we discuss the results from the simulations of the full spatial model, for which we assume that each cell is a continuous hexagon. We performed these simulations to ensure that our results on simplified geometries are not artefacts of the discretisation.

The systems of differential equations corresponding to the different versions of Model A are obtained from the reactions (1)–(10) by applying the law of mass action and linear diffusion of mobile components between neighbouring cellular compartments. Example equations can be found in Supporting Information S1.

### Model L

**Figure 3:**
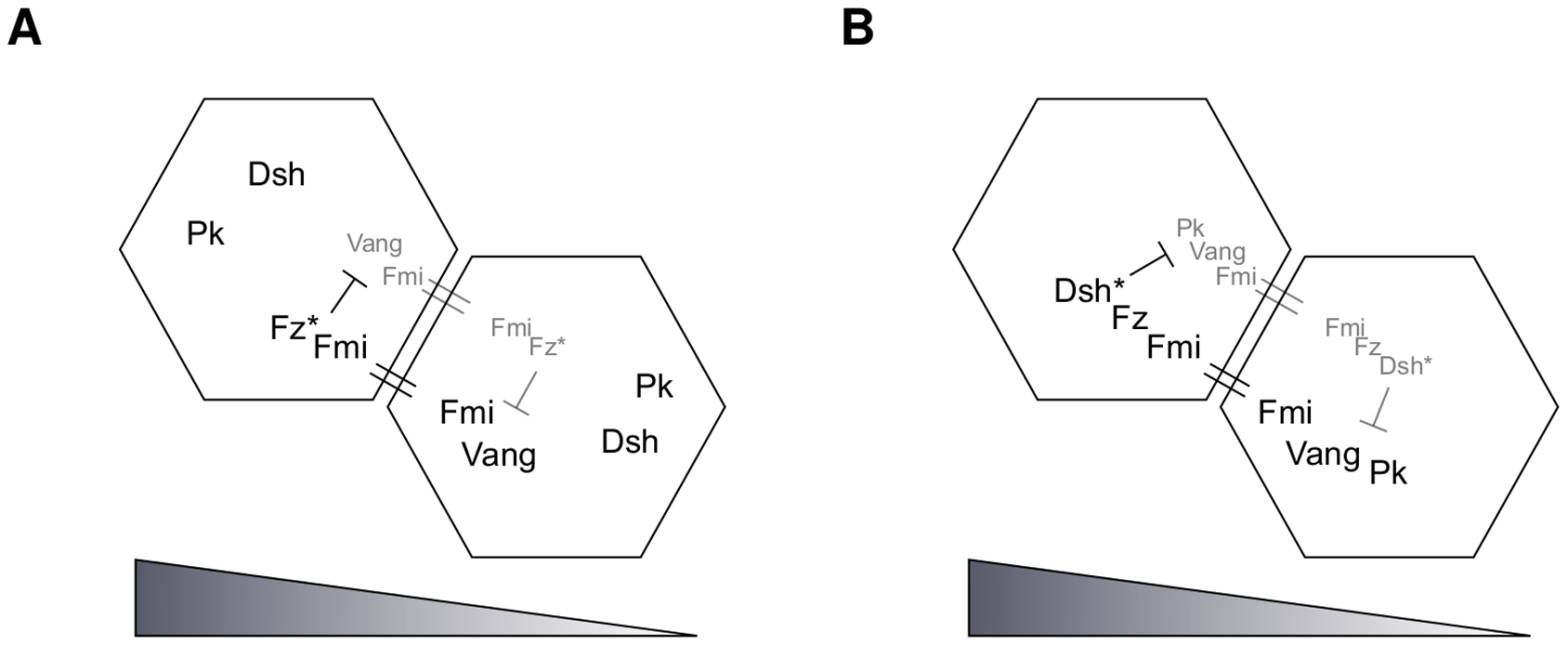
Feedback mechanism and global bias of Model L. Light grey represents a lower protein concentration than black. Binding over the cell membrane is indicated by = and T-signs represent inhibition. (A) First feedback loop: Fz* and its complexes inhibit the binding of Vang to Fmi; (B) second feedback loop: the Dsh* complexes inhibit the binding of Pk to Vang complexes (Dsh is phosphorylated when binding to the Fz*-ends of the cell bridging complexes, becoming Dsh*). The triangles at the bottom represent the tissue-scale ligand (and hence Fz*) gradient.



Model L incorporates the mechanism proposed by Le Garrec *et al.* in [8]. In this model, the global bias is provided by an initial tissue-wide ligand gradient, which is used up quickly by binding to Fz to give a tissue-wide gradient in ligand activated Fz*. The local amplification module relies on the feedback loops summarised in Figure 3. The interactions of the proteins and protein complexes can be described by the following reactions:

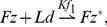
(11)

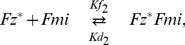
(12)

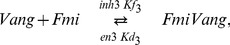
(13)

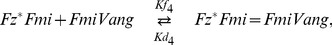
(14)

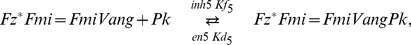
(15)

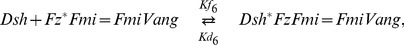
(16)

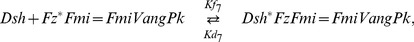
(17)

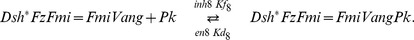
(18)


Ld represents a hypothetical ligand that binds to Fz, and Fz* denotes the bound (or ligand-activated) form of Fz. Dsh becomes phosphorylated on binding to the Fz*-ends of the cell bridging complexes, and is then denoted by Dsh*. The symbol = indicates complexes that bridge the membranes of two neighbouring cells. The forward reaction rates are 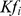 and the backward reaction rates are 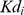 with 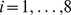. The two feedback loops are implemented by decreasing the forward reaction rates and increasing the backward reaction rates of equation (13) in response to the concentration of Fz* and Fz* complexes, and equations (15) and (18) in response to the concentration of Dsh* complexes. The factors are
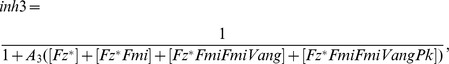


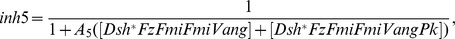


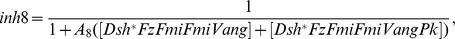
and



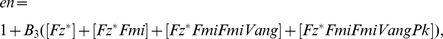


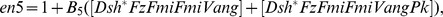


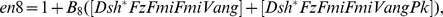
where 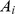 and 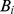 (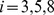) are positive constants.

As outlined above for Model A, we analyse this model for a one-dimensional and a two-dimensional discretisation (see Results section) and conduct simulations of the full spatial model (see Supporting Information S1, Section B.3). The systems of differential equations corresponding to reactions (11) – (18) are obtained by applying the law of mass action and linear diffusion of mobile proteins between neighbouring cellular compartments. Supporting Information S1 contains sample equations for each case.

## Results

Amonlirdviman *et al.* and Le Garrec *et al.* showed that their respective mechanisms, Model A and Model L, are capable of polarising a whole field of cells simultaneously. In both cases the results are based on numerical simulations of fields of two-dimensional hexagonal cells. The models have a common logical structure in that both consist of feedback mechanisms amplifying an imposed global bias. However, the basic mechanisms underlying these two components (imposed bias and local feedback) and their relative importance for the generation of coherent tissue-wide patterns of PCP are unclear. We addressed these issues by analysing the two models. The full models in two spatial dimensions are rather complex and do not lend themselves to a mathematical analysis very easily. Therefore, we discretised the systems in space and performed a computational analysis, systematically varying the parameter values and the initial conditions.

### The Persistent Global Bias Generates Polarity and Determines its Direction

We started our analysis by investigating the ability of the persistent imposed global bias to determine the final polarity in Models A and L. To this end, we initially reduced the models to one spatial dimension, applying the approach previously presented in [11]. We considered a line of two-sided cells with certain amounts of the proteins of interest on each side and intracellular diffusion between the two sides.

**Figure 4:**
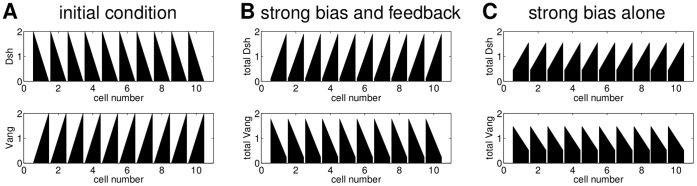
The persistent global bias has a strong impact on the final states of Model A. (A) Initial condition with a strong polarity of Dsh and Vang in the direction opposite to the direction of the final state observed in experiments. Pk and Fz are initially distributed homogeneously; (B) final state for a global bias of 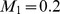 and the parameter values in Table S1; (C) final state for a global bias of 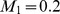, the parameter values in Table S1 but with no feedback (i.e. 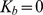). Even without feedback the cells polarise correctly, albeit more weakly than with feedback.

Model A relies on the interactions of the the four proteins Dsh, Fz, Vang and Pk. The persistent global bias is introduced as a decrease in the unbinding rate for Dsh from Fz and Fz containing complexes in the distal part of each cell. We simulated the model in Matlab for a row of ten cells with periodic boundary conditions and the parameter values in Table S1. As the readout, we present the final distributions of total Dsh and total Vang in each cell, which in each case include all relevant complexes. We find that the persistent global bias has a very strong impact on the final polarity. Figure 4 illustrates this result. To emphasise the effect of the persistent global bias we chose an initial condition with a strong global polarity for Dsh and Vang (shown in Figure 4A), opposite to the normal wild type distribution presented in Figure 1. Pk and Fz are initially distributed homogeneously in every cell, and there are no complexes.

Figure 4B shows the final state for a strong global bias. The polarity is reversed compared to the polarity of the initial conditions. Weaker global bias yields weaker polarity, but in the same direction (not shown). To compare these results with a final state that relies only on the persistent global bias we performed the simulation with the same initial condition and the same strength of the global bias but with the feedback loop switched off, i.e. setting 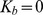. This yields a weaker polarity (Figure 4C) but its direction is still reversed compared to the direction of the initial condition in Figure 4A. Thus, with the persistent global bias Model A generates polarity in the direction of the bias irrespective of the initial conditions and the feedback loop.

For their simulations of Model L, Le Garrec *et al.* used a stochastic approach [8]. To make the results comparable to those for Model A, we used a deterministic approach instead, ensuring that we get results which agree with [8] (see Supporting Information S1 as well as Table S4 and Figure S2). While in Model A the persistent global bias is introduced at the level of single cells, Model L relies on a tissue-wide gradient of a ligand (Ld) for Fz. To investigate the contribution of the gradient to polarity we again assumed a row of two-sided cells with certain concentrations of the six proteins Ld, Dsh, Fz, Pk, Vang, Fmi and their complexes on each cell side. As initial conditions we chose a gradient of the ligand while all other proteins are distributed homogeneously within the cell. Initially, there are no complexes.

**Figure 5:**
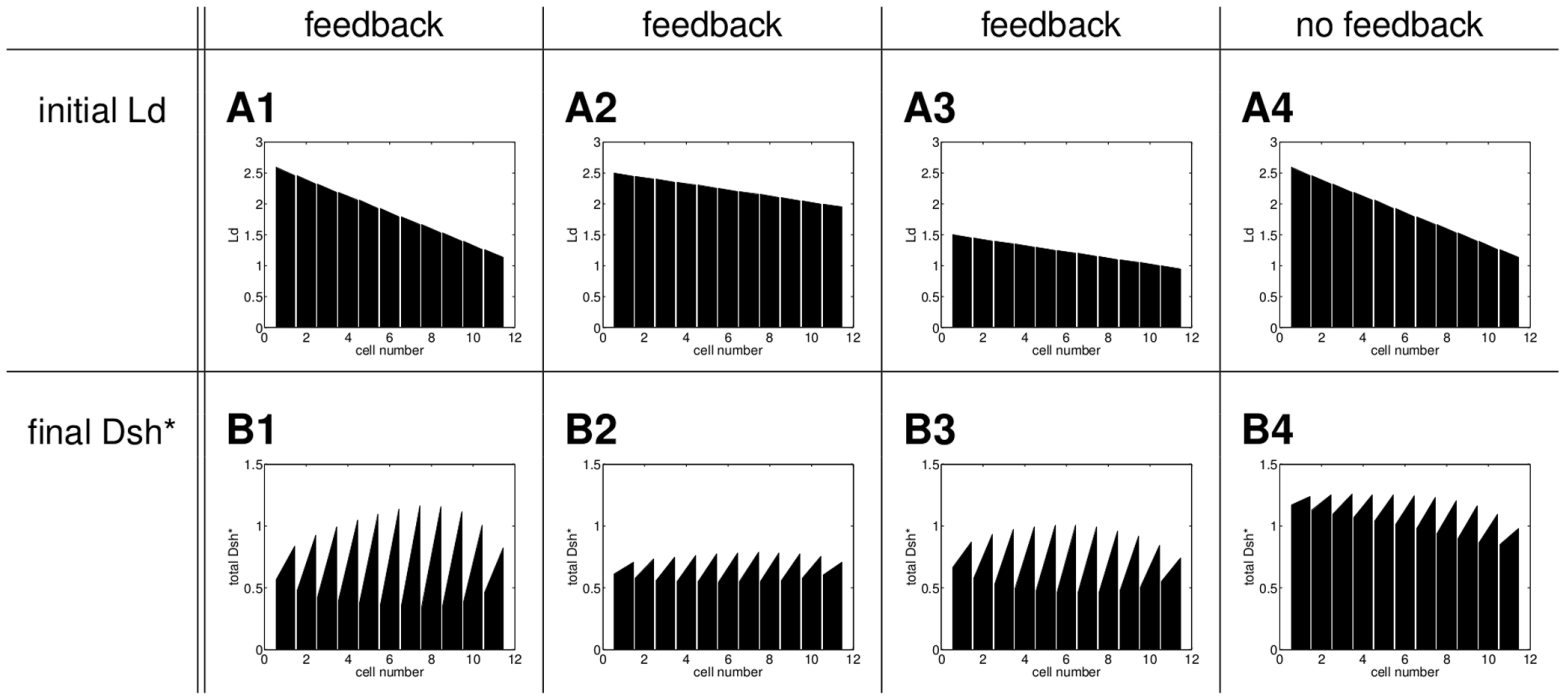
Effect of the initial ligand gradient on the final Dsh* distributions of Model L with and without feedback. Column 1: The weaker polarity in the first and the last cell of the row is due to the boundary conditions. The polarity in the left half of the row is weaker because in cells 1–3 there is more ligand than Dsh, Vang and Pk; column 2: a shallow gradient with high levels of Ld gives weak polarity; column 3: for a shallow gradient with lower levels of Ld we get stronger polarity; column 4: the same initial gradient as in A1 and no feedback (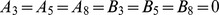) yields weaker polarity than in B1. The parameter values are shown in Table S4.

The main results of our analysis are illustrated in Figure 5. Row A shows different initial ligand gradients and row B displays the corresponding final distribution of the sum of the Dsh* complexes as a readout, since in [8] this is assumed to determine the direction of the hair growth; the hairs are assumed to grow at the end of the cell with the highest Dsh* concentration. For the simulations we considered eleven cells plus two boundary cells, one at each end of the row (cells 1 and 13). Let 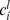 and 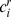 be the concentrations of any protein or protein complex in cell 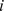 on the left and the right side, respectively. For the boundary cells we assume 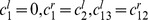 and 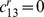 as well as no intracellular diffusion. The remaining interactions in these cells are governed by the same equations as for the rest of the cells.

In Figure 5 A1 we assume a decreasing linear ligand gradient. This initial gradient yields polarity to the right with weaker polarity at the ends of the row due to the boundary conditions (see Figure 5 B1). The strength of polarity depends on the slope of the gradient such that a shallower gradient yields weaker polarity as shown in Figure 5 column 2. Furthermore, the amount of Ld relative to the amounts of the other proteins is important. In Figure 5 A2 the total amount of Ld in each cell is higher than for Vang, Dsh or Pk. The initial gradient in A3 has the same slope as the gradient in A2 and less Ld in each cell than any of the other proteins. This yields a stronger polarity as shown in Figure 5 B3. In all cases the polarity is generated by the ligand gradient and the feedback determines the strength of polarity. This is shown in column 4, where we chose the same initial ligand gradient as in column 1 and no feedback. Compared to B1, the final state in B4 displays a weaker polarity but still in the same direction.

The direction of the final polarity is determined by the direction of the initial ligand gradient. An increasing gradient would yield polarity to the left. Furthermore, the results do not depend on the type of gradient. E.g. a decreasing exponential gradient or an initial gradient in which the initial amount of ligand is the same on both sides of each cell would yield similar final states (not shown).

These results demonstrate that both the slope and the total amounts of Ld in the initial gradient determine the strength of polarity, while the direction of polarity depends on the direction of the gradient. Furthermore, while the initial gradient is sufficient to establish polarity, the feedback loops are required to enhance the strength of polarity.

Our analysis shows that in Model A the cell-intrinsic bias itself is persistent while in Model L the initial ligand gradient is used up very quickly to generate a persistent gradient in Fz* by binding to Fz. Both biases exhibit the same mechanistic behaviour as they prevent any homogeneous unpolarised steady states and determine the direction of the final polarity. In a system with such a persistent global bias, the role of the feedback is to regulate the strength of polarity.

### Without a Persistent Global Bias the Feedback Loop can Generate Polarity

In the previous section, we have established that in the presence of a persistent global bias the feedback loop influences the strength of polarity but not its generation or direction. As a next step, we analysed the capabilities of the feedback on its own. To this end, we considered the behaviour of the models if there is no persistent global bias.

Without the persistent global bias, for initial conditions in which all the proteins are distributed homogeneously and there are no initial complexes, neither of the models can generate polarity (not shown). Therefore, we assume a small initial imbalance in each cell in one of the proteins. The remaining proteins are distributed homogeneously and initially there are no complexes. in contrast to the persistent global bias, such a transient initial imbalance does not prevent the establishment of homogeneous unpolarised steady states.

**Figure 6:**
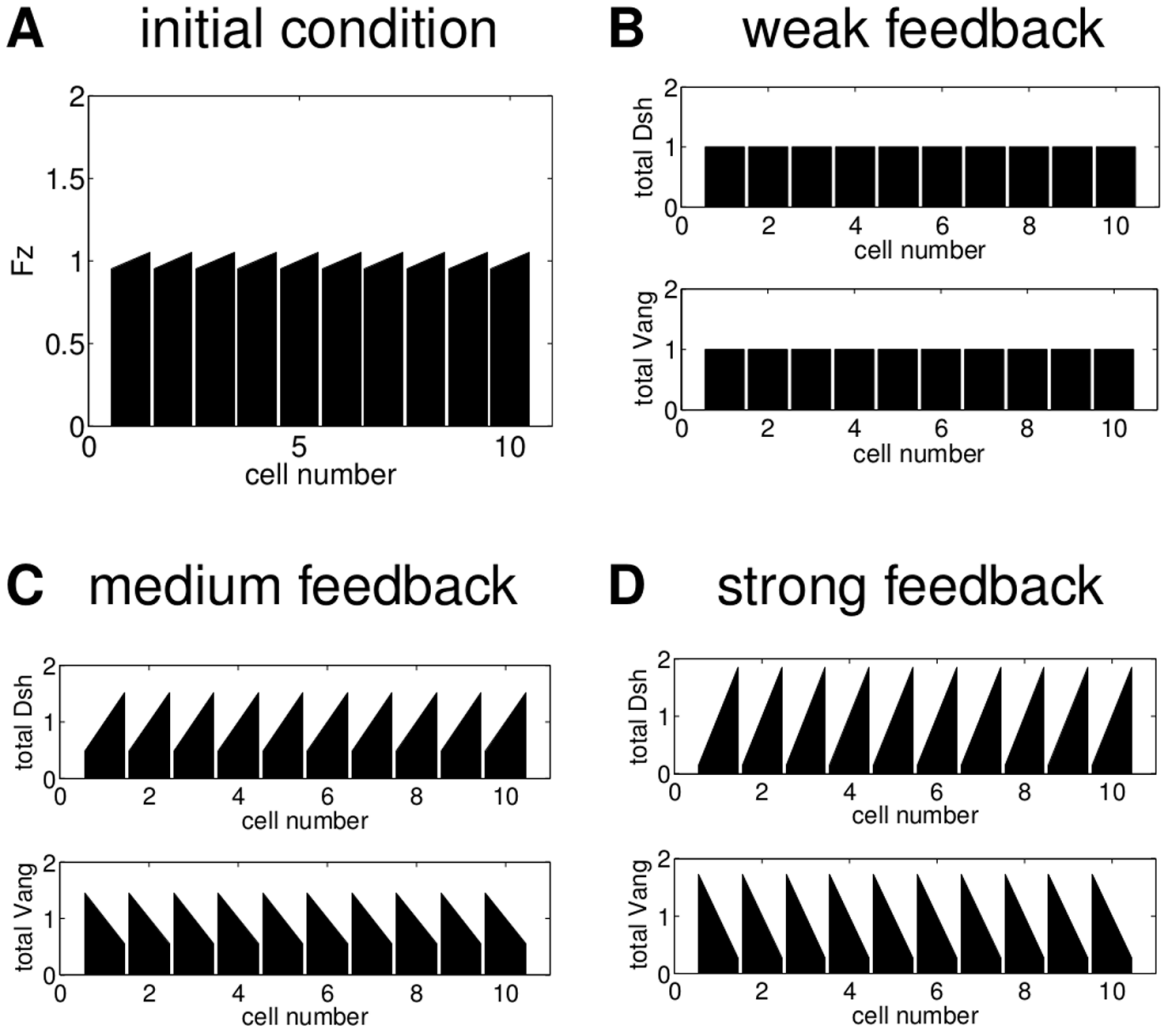
Final states of Model A for different strengths of feedback with no imposed global bias. (A) Initial condition: an imbalance in Fz with a difference of 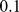 between right and left side; initially, the other proteins are distributed homogeneously; (B) final distribution of total Dsh and total Vang for a weak feedback with the corresponding parameter values 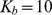 and 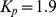, (C) final distribution of total Dsh and total Vang for a stronger feedback with 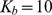 and 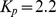, D final distribution of total Dsh and total Vang for an even stronger feedback with 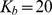 and 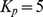. All other parameter values as listed in Table S1.

For Model A we considered a biologically motivated small initial increase in Fz in every cell as shown in Figure 6A. Figures 6B–D show the behaviour of Model A for different strengths of feedback and no persistent global bias. If the feedback is weak, a homogeneous unpolarised steady state arises (Figure 6B). Increasing the strength of the feedback yields polarity and an even stronger feedback increases the strength of polarity (Figure 6C and D).

**Figure 7:**
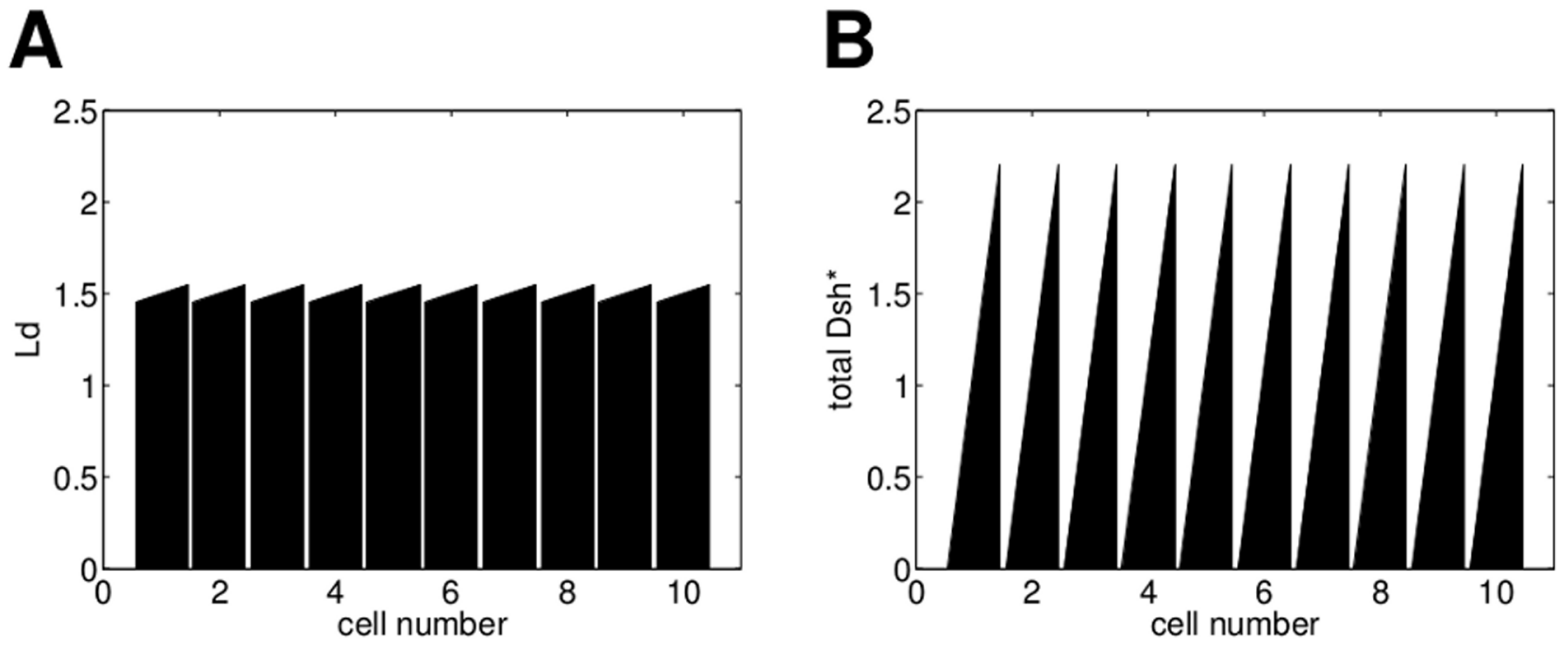
For a small initial Ld imbalance in each cell Model L can yield polarity. (A) Initial ligand distribution with a small imbalance in every cell, the difference between left and right in each cell is 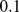. Initially, the other proteins are distributed homogeneously and there are no protein complexes. (B) Final state of total Dsh* for the parameter set in Table S5.

For Model L we considered an initial imbalance in Ld. The initial condition and the corresponding final state for a row of ten cells are shown in Figure 7. The total amount of ligand in each cell is less than the total amount of any other protein to ensure that we do not get weaker polarity caused by excessive amounts of Ld as observed in the previous section (Figure 5). Figure 7B shows the final Dsh* distribution for periodic boundary conditions and a set of parameter values which were optimised to get polarity (see Supporting Information S1, Figure S3 as well as Tables S5 and S6).

These results demonstrate that in both models a persistent global bias is not required for the generation of polarity when there is an initial imbalance that can be amplified by a sufficiently strong feedback.

**Figure 8:**
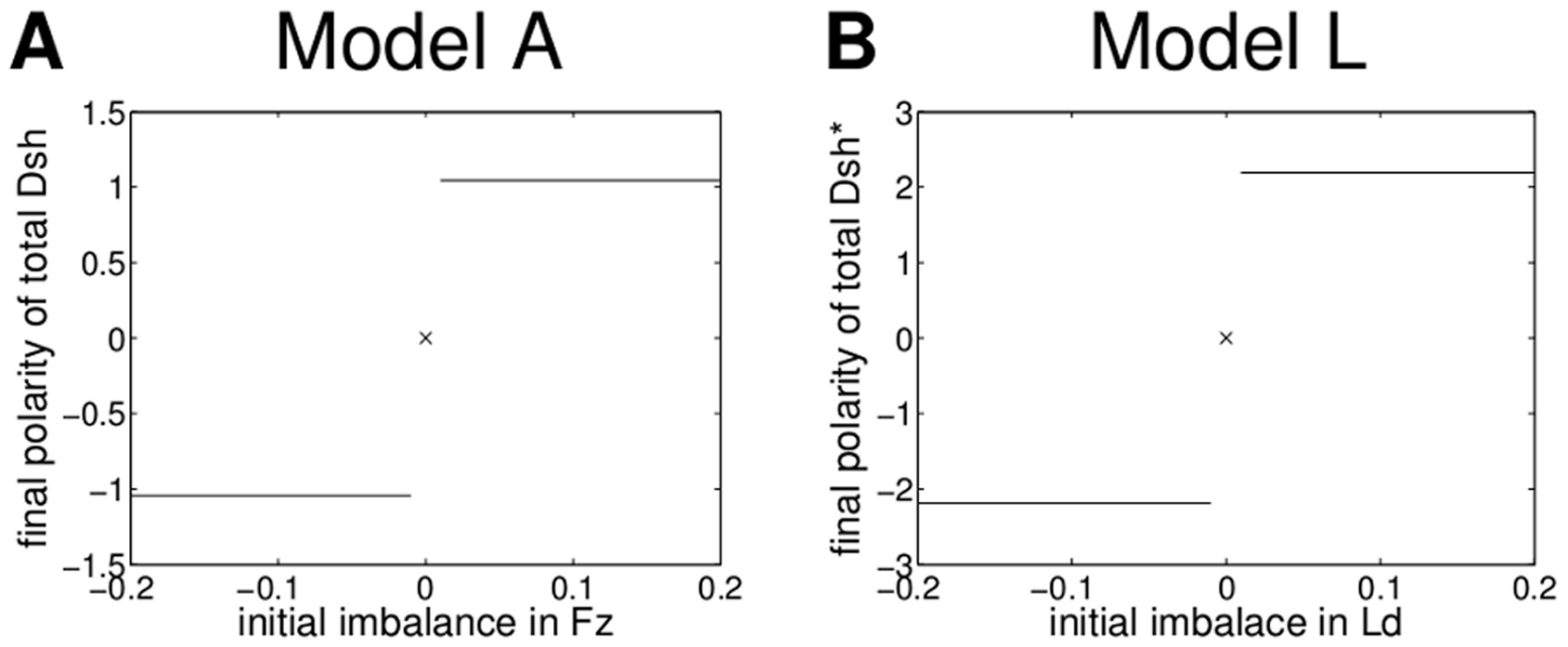
Effect of the initial imbalance on the final polarity of Models A and L - without a persistent global bias. We consider a row of ten cells with identical initial conditions. The strength of the initial imbalance is determined by the difference between the right and left sides of a cell in Fz for Model A and Ld for Model L, respectively; in each case the other proteins in the model are initially distributed homogeneously and there are no complexes. (A) The strength of the final polarity of Model A as the difference in total Dsh between the right and left sides of a cell. The parameter values are shown in Table S1. (B) The strength of the final polarity of Model L as the difference in total Dsh* between right and left side of a cell. We used the parameter values in Table S5. For both models even the smallest non-zero perturbations are amplified, due to the instability of the unpolarised state to polarised perturbations.

To analyse the impact of the initial imbalance on the final polarity, we varied its strength from −0.2 (imbalance to the left) to 0.2 (imbalance to the right). Figure 8 shows the final strength of polarity dependent on the strength of the initial imbalance for Models A and L. For both models a small initial imbalance is necessary to break symmetry, and the direction of the polarity of the final state depends on the direction of the imbalance in the initial conditions.

These results show that in both models the feedback mechanism alone can generate bistability across the membranes of two neighbouring cells, and thereby amplify small initial imbalances in protein distribution to generate strong cellular polarity. Therefore, without the imposition of a persistent global bias, Models A and L provide specific examples of the feedback and diffusion model class described in [11].

Our discretised analysis in one spatial dimension has shown that although the two models differ in their molecular details, the mechanisms at the core are similar. In both models, the global cue ensures polarity and determines its direction, while the feedback mechanism controls the strength of the polarisation. In the absence of the global cue the feedback mechanism can yield an unpolarised state or polarity depending on the parameter values and the initial condition. For certain parameter values the unpolarised steady state is unstable to polarised perturbations and a small initial imbalance is amplified. The direction of polarity depends on the direction of the initial condition.

**Figure 9:**
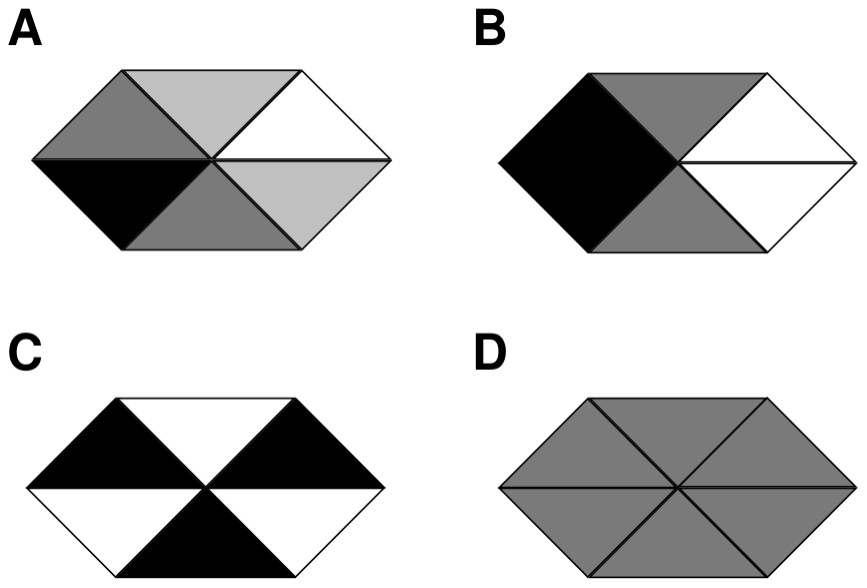
In two spatial dimensions four different types of steady states can occur. (A) a side polarised configuration, (B) a vertex polarised configuration; (C) a triangular state: (A) and (B) are possible with six directions and (C) with two distinct orientations. (D) unpolarised configuration.

As a next step we determined the extent to which these results are valid for hexagonal cells. While in two-sided cells in one spatial dimension there is only one type of inhomogeneous steady state, in two spatial dimensions with hexagonal cells we have to distinguish between vertex polarity, side polarity and a triangular state as illustrated in Figure 9. Since in the wild type *Drosophila* wing the hairs grow from the most distal tip of approximately hexagonal cells, we are mainly interested in vertex polarity (Figure 9B).

In the next section, we extend our analysis of the two models to hexagonal cells in two spatial dimensions. The persistent global cues yield only vertex polarity, since they impose symmetry constraints which are inconsistent with side polarity and the triangular states. Therefore, we omit global cues and investigate which steady states the feedback mechanisms alone can generate and whether they are stable.

### Without the Global Bias Vertex Polarity is Unstable

To analyse the models in two spatial dimensions, we assume hexagonal cells, which are divided into six compartments. Each compartment contains a certain amount of the proteins and protein complexes. Diffusion occurs between a compartment and its two neighbouring compartments in the same cell. Details about the systems of equations are given in Supporting Information S1. For the numerical analysis we simulated the system for one cell, applying periodic boundary conditions for the intercellular binding. Hence, our domain represents an infinite field of hexagonal cells with the same initial conditions.

**Figure 10:**
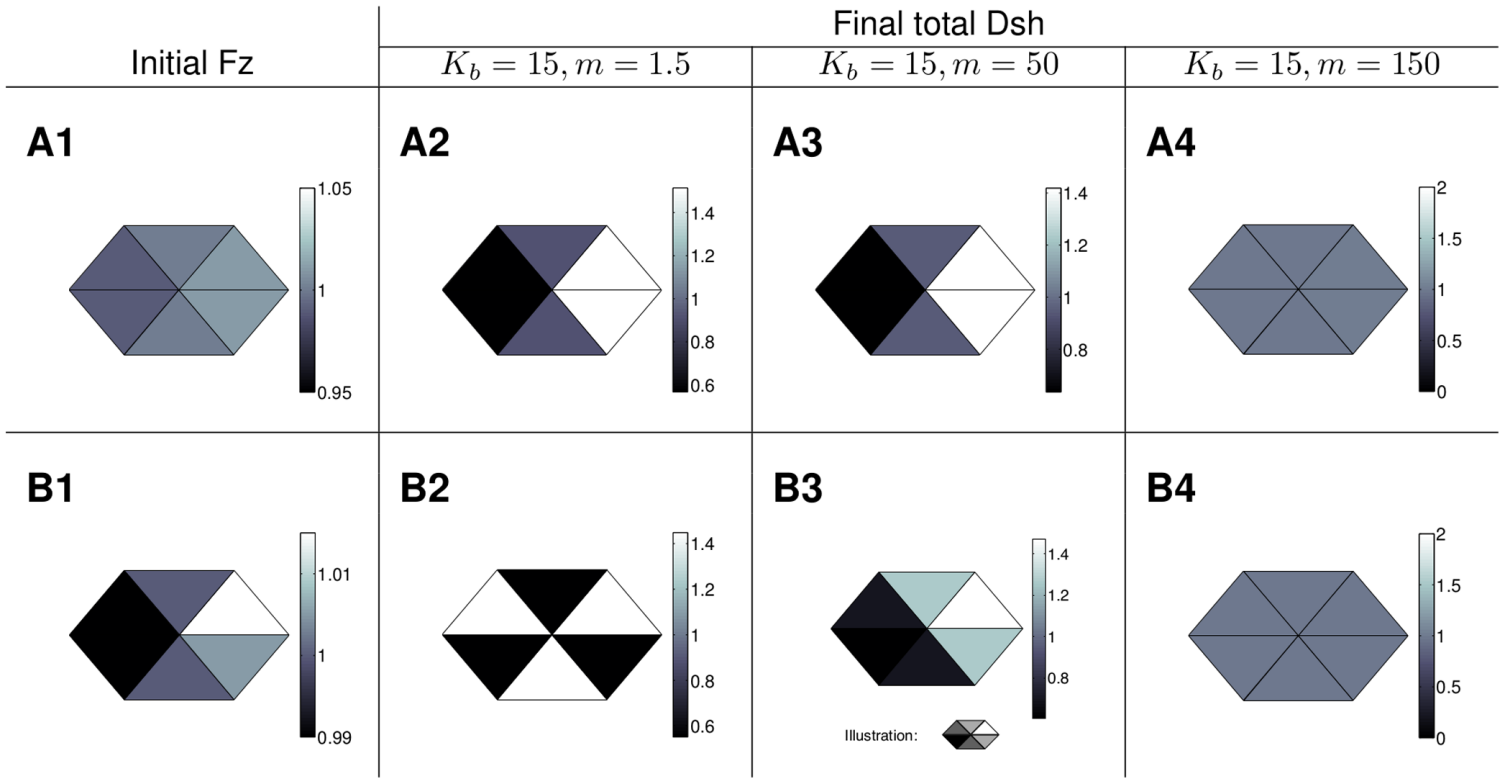
Examples of the steady states of Model A in a periodic array of hexagonal cells. Top: Initial Fz distribution with a slight vertex polarity and final total Dsh distribution for a fixed feedback (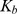) and different values of the diffusion strength (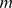). Bottom: Inhomogeneous initial Fz distribution (B1, note the different scale compared to A1 to highlight the slight inhomogeneity) and final total Dsh distributions for a fixed feedback strength (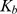) and different values of the diffusion coefficients (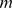). The remaining parameter values can be found in Table S2. Columns 2 and 3: For these parameter values vertex polarity is not robust to noise in the initial Fz distribution. Column 4: For sufficiently strong diffusion both initial conditions yield the unpolarised steady state.

Figure 10 shows the behaviour of Model A in two spatial dimensions for two different initial distributions of Fz. The other proteins are initially distributed homogeneously and there are no initial complexes. As illustrated in row A of Figure 10, for a certain feedback strength and sufficiently slow diffusion (for details on parameter values see Table S2), a weak initial vertex polarity in Fz is amplified to a final state with a strong vertex polarity in total Dsh. For the same parameter values, an initial condition with an inhomogeneous Fz distribution however yields triangular final distribution of total Dsh in Figure 10B2 or the side polarised state in B3 depending on the strength of diffusion. This indicates that, for this choice of parameter values, the vertex polarised state exists but is unstable. Increasing the diffusion strength and thereby the coupling of the compartments within the cell yields the homogeneous unpolarised state for both initial conditions as shown in column 4 of Figure 10. For Model L we observe a similar behaviour (see Figure S4).

To extend these results to a wider range of parameter values we performed a parameter scan, systematically varying the diffusion coefficients and the strength of the feedback. For every steady state, we calculated the eigenvalues of the corresponding linearised system to determine its stability.

**Figure 11:**
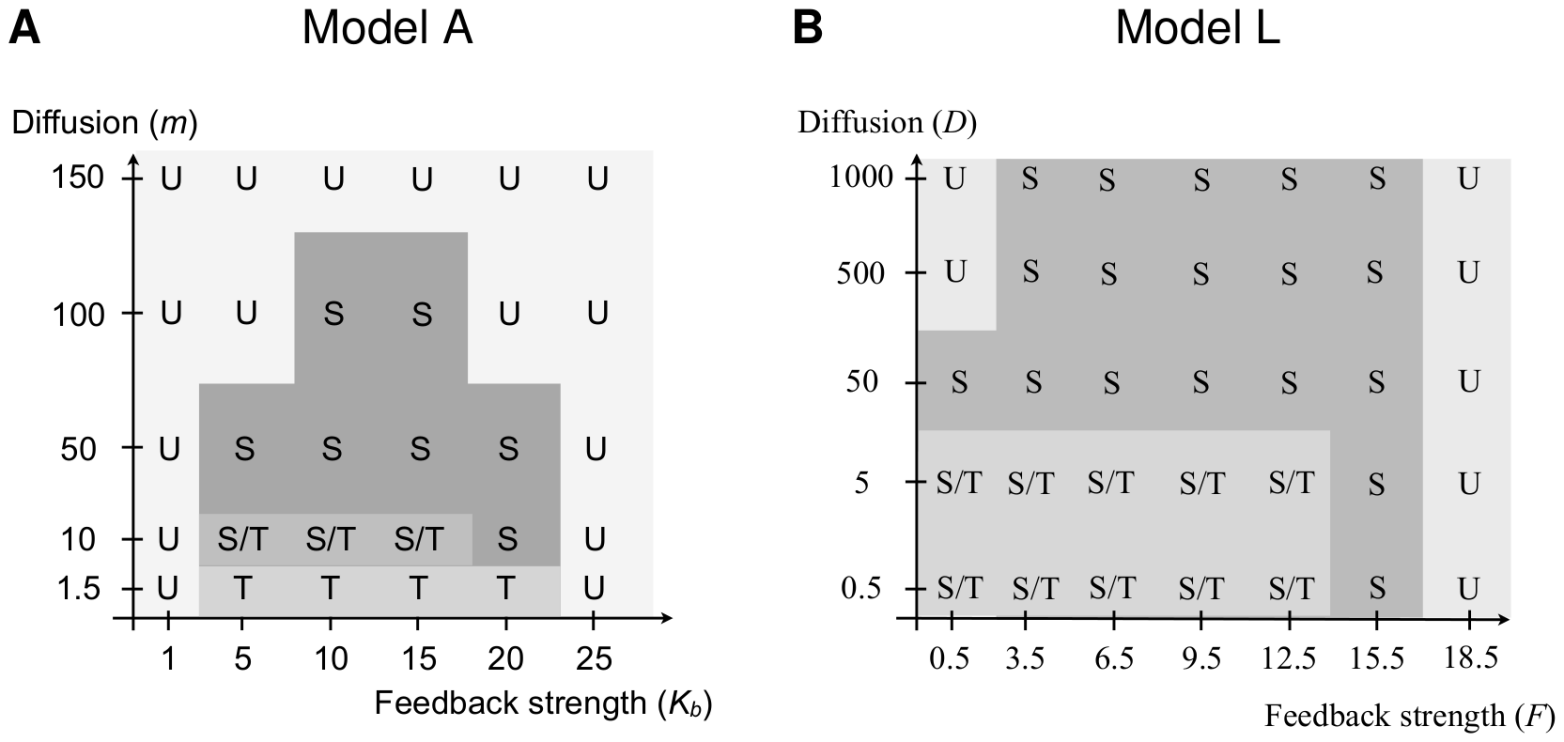
Stable steady states for different parameter combinations. The letters indicate the steady states which are stable for a certain parameter combination, U - unpolarised, S - side polarised, T - triangular, S/T-bistable. The values for the feedback strength were chosen to cover all possible behaviour. The parameter values for Model A are shown in Table S2; the parameter values for Model L in Table S7. A vertex polarised steady state is never stable.

Depending on the parameter values, we found that either the unpolarised steady state, the side polarised state in one of the six directions or a triangular state is stable. Maps of the results from the parameter scan for the two models are shown in Figure 11.

For Model A, within the range of our parameter scan, intermediate feedback strength combined with weak intracellular diffusion yield the triangular state. For higher diffusion the system tends to a side polarised state and the direction of polarity depends on the direction of the initial condition. Increasing diffusion further yields the unpolarised state. The vertex polarised state was never stable in our parameter scan.

For Model L, within the range of our choice of parameter values (see also Table S7), a low diffusion yields the triangular state while increasing the diffusion coefficients evokes a side polarised state in a direction determined by the initial condition. In contrast to Model A, side polarity is stable if the diffusion coefficient is increased by several orders of magnitude. For sufficiently large diffusion, Model L will also yield the unpolarised state. For smaller values of the diffusion coefficient, the unpolarised state occurs if the feedback strength is sufficiently high or sufficiently low. Again, the vertex polarised state was never stable in our parameter scan.

This analysis gives strong evidence that for both models the vertex polarised state can exist, but when it does, it is always unstable. Due to the complexity of the models we cannot completely rule out the existence of a stable vertex polarised state. To ensure that this result is not an artefact of our discretisation we numerically approximated the solution of the full spatial models applying the finite element method. We considered different domains for the proteins that diffuse in the whole cell, proteins and complexes that only diffuse in the membrane and cell bridging complexes that are restricted to the edge of the membrane common to the cells they link together. We find that, for both models, vertex polarity is unstable to asymmetric perturbations (see Supporting Information S1, Tables S3 and S8 as well as Figures S1 and S5 for more details).

## Discussion

Planar cell polarity relies on the coordination of three modules: a global module that links the polarity of the individual cells to the tissue axis, a local module which ensures alignment of neighbouring cells and a readout module that processes the polarity and ensures correct alignment of extracellular structures like hairs or bristles. To improve our understanding of how the first two modules could interact to establish planar cell polarity we conducted a numerical analysis of Model A (adapted from [7]) and Model L (adapted from [8]). Although with a numerical analysis it is not possible to analyse the infinite range of all possible parameter values, we are confident that due to the methods applied and the extent of our investigations we have not missed any essential properties of the models.

As combinations of a global cue and local feedback mechanisms, the Models A and L share a similar logical structure. Although their molecular details are different, our analysis has revealed that they exhibit a common behaviour, indicating a common mechanism at their core.

We found that both models incorporate the global module as a persistent global cue which for any parameter set generates polarity and determines its direction. In one spatial dimension we obtained polarity to the right or the left, while in two spatial dimensions hexagonal cells exhibited vertex polarity, the distribution of the core proteins characteristic for planar cell polarity in the *Drosophila* wing.

In the literature, several candidates for persistent global biases have been proposed. The ligand gradient in Model L is based on the idea of a gradient of a putative ‘Factor X’, which is assumed to be a ligand for Fz but which has not been identified so far (reviewed in [12]). Another possibility is that the global module relies on the transmembrane proteins Dachsous (Ds) and Fat (Ft) and the cytoplasmic protein Four-Jointed, which are expressed in tissue wide gradients and interact to generate intracellular gradients, which might be read by the local module or the readout module (reviewed in [4]). Alternatively, work by Aigouy *et al.* [13] in the *Drosophila* wing suggests that the global cue is mechanical. They find that, due to the contraction of the hinge, wing cells are subject to anisotropic tension that regulates their alignment.

Model A incorporates an intracellular persistent global cue, such that each cell is already polarised. Such a global cue could be generated by polarised transport along a polarised network of microtubules [14]. In addition to the cytoskeleton, the plasma membrane has been found to be involved in the polarisation of cells. In [15], Simons *et al.* show that the recruitment of Dsh by Fz and their interactions are dependent on the local pH and charge of the membrane.

Since all these possible mechanisms for the global module are examples of persistent global biases, it is very likely that such a mechanism plays a role in generating long range coordinated polarity. The details of the local module are more elusive. Models A and L propose two detailed feedback mechanisms based on interactions of the core proteins. We have analysed the behaviour of these feedback mechanisms in the absence of the persistent global biases. For both models we found that they yield a homogeneous unpolarised state or inhomogeneous polarised states depending on the parameter values and the initial conditions. In one spatial dimension, the inhomogeneous states arise from small initial imbalances in the cells and are polarised to the right or the left depending on the direction of the initial condition. In two spatial dimensions for hexagonal cells we obtained stable side polarity in six directions or stable triangular states, depending on the feedback and diffusion strength. Increasing diffusion increases the coupling within a cell and yields the transition from a triangular state to side polarity. Vertex polarity however is always unstable. Thus, to generate robust vertex polarity that is insensitive to noise both models require a persistent global bias.

These results indicate that the feedback loops in Models A and L share common mechanistic details. In both models the feedback loops regulate intercellular binding between proteins and complexes of two compartments of neighbouring cells. The stability of side polarity and the triangular state together with the instability of vertex polarity suggests that the feedback mechanisms introduce a bistable switch across membranes of neighbouring cells. Bistability ensures that states with different protein levels in adjacent compartments are stable, i.e. side polarity and the triangular state. For vertex polarity the top and bottom compartment in the cell would have different amounts of proteins and complexes as their neighbours within the same cell but the same amount as their neighbours in the neighbouring cell (see Figure 9). Hence, vertex polarity would require a tristable system which enables the stability of states with a combination of adjacent compartments with the same protein level and adjacent compartments with different protein levels.

The mechanism of Models A and L is clearly distinguishable from the feedback loop proposed by Burak and Shraiman in [16]. In their model they combine a local bistable switch across membranes with a non-local inhibition within a cell. Thereby, the mechanism not only segregates protein complexes across the membrane but also within a cell. Hence, it is capable of generating robust vertex polarity in the absence of a persistent global bias.

Taken together the main open question is whether the feedback loop can coordinate polarity across tissues independent of the global bias or whether it is really just a bistable switch which introduces an amplification mechanism.

## Supporting Information

Figure S1 **Initial conditions and final states for simulations of the full spatial version of Model A.** Proteins and protein complexes presented occur only on the membrane. In each case a line plot and a two-dimensional representation are shown. Table S3 shows the chosen parameter values. Row A: a weak initial vertex polarity in Vang yields vertex polarity. However, this state is not stable to perturbations that break the anterior-posterior symmetry. Row B: an initial condition with a side polarity yields side polarity; the line plots show top and bottom half of the membrane separately.
(TIF)

Figure S2 **Model L gives similar result to the original model in** [**8**] **.** (A) Initial imposed ligand gradient from [8], adopted to our geometry; (B) final Dsh* distribution from a deterministic simulation for the parameter values in [8] (see Table S4). The weaker polarity in the first and last cell of the row is due to the boundary conditions.
(TIF)

Figure S3 **Model L does not polarise without a global bias even for a strong initial ligand imbalance in every cell and the parameter values in Table S4.** (A) Initial ligand distribution with a strong polarity, (B) Dsh* distribution at an intermediate time point, (C) final Dsh* distribution.
(TIF)

Figure S4 **Examples of the steady states of Model L in a periodic array of hexagonal cells.** Top: initial Ld distribution with a slight vertex polarity and final total Dsh* distributions for a fixed feedback strength and different values of the diffusion coefficients. bottom: Inhomogeneous initial Ld distribution (B1, note the different scale compared to A1 to highlight the slight inhomogeneity) and final total Dsh* distribution for a fixed feedback strength and different diffusion strength. The remaining parameter values are presented in Table S7. Columns 2 and 3: For these parameter values vertex polarity is not robust to noise in the initial Ld distribution. Column 4: For sufficiently strong diffusion both initial conditions yield the unpolarised steady state.
(TIF)

Figure S5 Final distribution of total Dsh* for the full spatial version of Model L for different initial conditions. Protein and protein complex distributions occur on the membrane. In every case a line plot and a two-dimensional plot are shown. The corresponding parameter values are shown in Table S8. Row A: an initial ligand distribution that is weakly vertex polarised yields vertex polarity of total Dsh*. However, this state is not stable to perturbations that break the anterior-posterior symmetry. Row B: an initially side polarised ligand distribution yields a side polarised distribution of total Dsh*. The line plots show top and bottom half of the cell separately.
(TIF)

Table S1 **Set of parameter values for which Model A, exemplified by equation (S1), can generate polarity with and without the global bias.** We used these parameter values for the simulations shown in Figures 4, 6 and 8 in the main text.
(PDF)

Table S2
**Choice of parameter values which were used for the parameter scan for the two dimensional, compartmentalised version of Model A in the main text.** The values of the remaining parameters are the same as in Table S1. The diffusion depends on a parameter 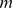 that was varied to gain insight into the effect of the speed of diffusion on the final state. The parameter 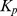 is varied to investigate the influence of the feedback strength on the steady states. These parameter values were used to obtain Figures 10 and 11 in the main text.(PDF)

Table S3 **Set of parameter values for simulations of Model A in Figure S1.**
(PDF)

Table S4 **Set of parameter values from** [**8**] **after rescaling of the diffusion coefficients.** We used these values for the simulations of Model L in Figures S2 and S3 as well as in Figure 5 in the main text.
(PDF)

Table S5
**Parameter values for which Model L can have a polarising instability in the absence of a ligand gradient.** Entry 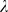 represents the maximal real part of the eigenvalues of the system exemplified by equation (S5). The corresponding eigenvector is given in Table S6. These parameter values were used to generate Figures 7 and 8 in the main text.(PDF)

Table S6
**For Model L the parameter values in Table S5 can give polarised distributions of the protein complexes.** Eigenvector corresponding to the eigenvalue 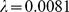 for the homogeneous unpolarised steady state of the system exemplified by equation (S5) and the parameter values in Table S5. To reduce the computational effort we applied the conservation laws for the six proteins and therefore there are no entries corresponding to 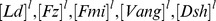 and 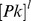.(PDF)

Table S7
**Choice of parameter values for Model L with variable feedback and diffusion strength.** The parameter values for the diffusion coefficients from Table S5 are multiplied by a parameter 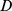. This was varied in the parameter scan for the two dimensional compartmentalised version of Model L in the main text to gain insight into the effect of the speed of diffusion on the final state. The feedback parameters 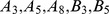 and 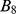 from Table S5 were all multiplied by the same parameter 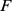 and 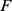 was varied to investigate the influence of the feedback on the final state. The parameter values for 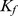 and 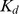 are the same as in Table S5. This parameter set was used to generate Figure S4 as well as Figure 11 in the main text.(PDF)

Table S8 **Parameter values for the simulations of Model L, exemplified by (S7), in Figure S5.**
(PDF)

Supporting Information S1 **Example equations for the different discretisations of Models A and L as well as parts of the analysis that are beyond the scope of the main text.**
(PDF)
