# Supplementary figures and images for: Is a Persistent Global Bias Necessary for the Establishment of Planar Cell Polarity?
Source: PLoS One. 2013 Apr 8;8(4):e60064. doi: 10.1371/journal.pone.0060064 (PMC3620226; doi:10.1371/journal.pone.0060064)

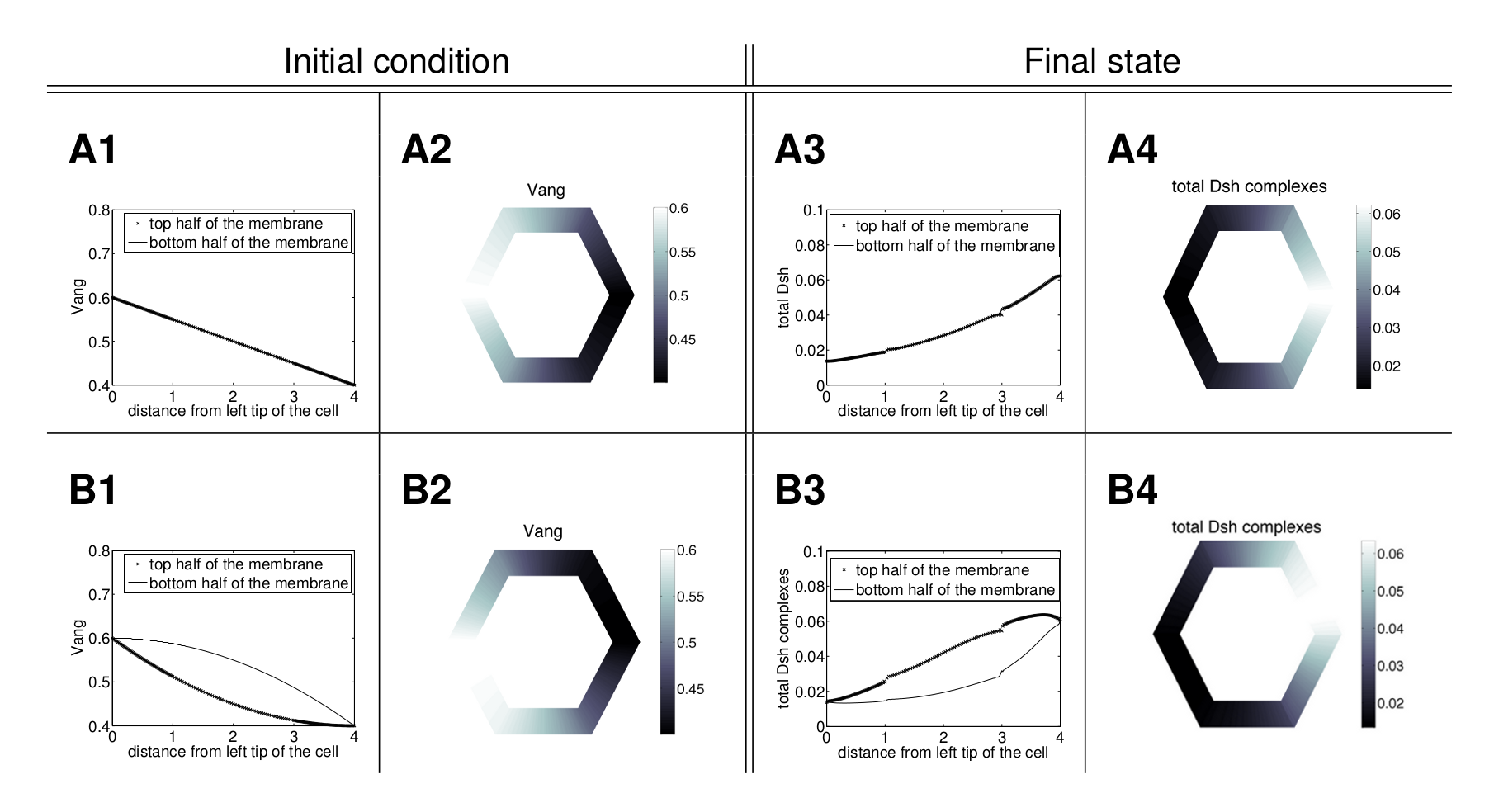

Supplement: Figure S1 — Initial conditions and final states for simulations of the full spatial version of Model A. Proteins and protein complexes presented occur only on the membrane. In each case a line plot and a two-dimensional representation are shown. Table S3 shows the chosen parameter values. Row A: a weak initial vertex polarity in Vang yields vertex polarity. However, this state is not stable to perturbations that break the anterior-posterior symmetry. Row B: an initial condition with a side polarity yields side polarity; the line plots show top and bottom half of the membrane separately. (TIF) [file pone.0060064.s001.tif]

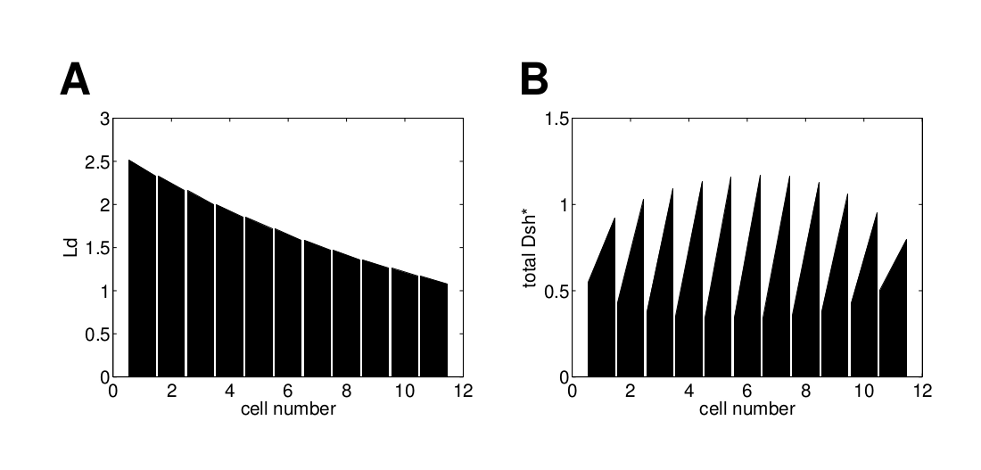

Supplement: Figure S2 — Model L gives similar result to the original model in [8] . (A) Initial imposed ligand gradient from [8], adopted to our geometry; (B) final Dsh* distribution from a deterministic simulation for the parameter values in [8] (see Table S4). The weaker polarity in the first and last cell of the row is due to the boundary conditions. (TIF) [file pone.0060064.s002.tif]

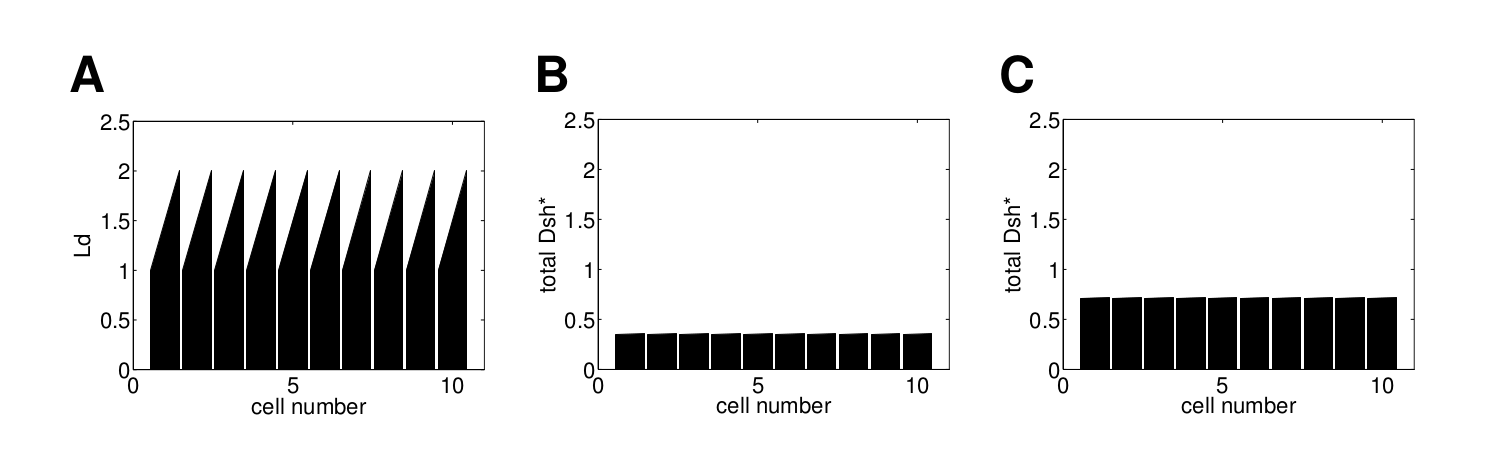

Supplement: Figure S3 — Model L does not polarise without a global bias even for a strong initial ligand imbalance in every cell and the parameter values in Table S4. (A) Initial ligand distribution with a strong polarity, (B) Dsh* distribution at an intermediate time point, (C) final Dsh* distribution. (TIF) [file pone.0060064.s003.tif]

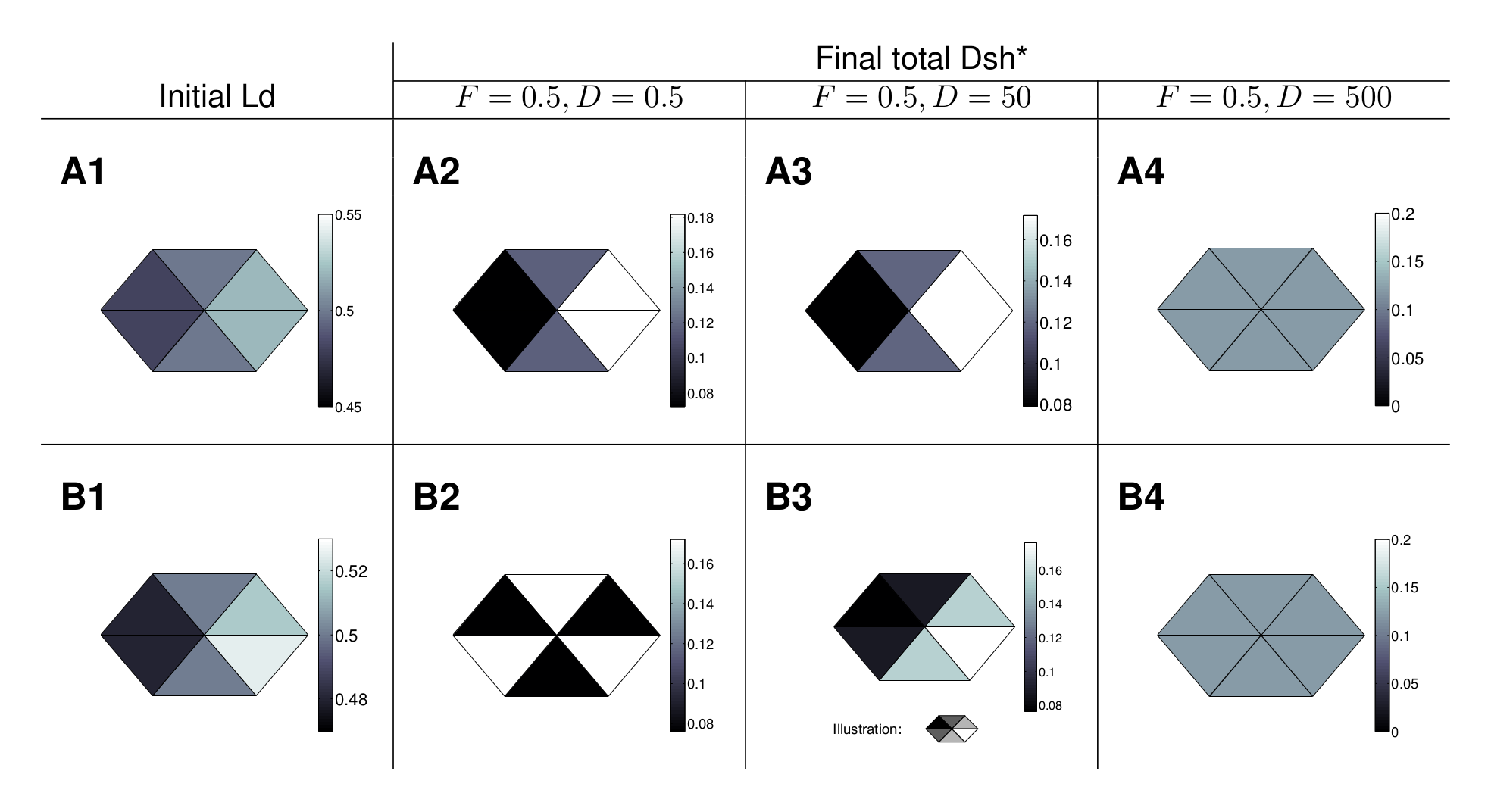

Supplement: Figure S4 — Examples of the steady states of Model L in a periodic array of hexagonal cells. Top: initial Ld distribution with a slight vertex polarity and final total Dsh* distributions for a fixed feedback strength and different values of the diffusion coefficients. bottom: Inhomogeneous initial Ld distribution (B1, note the different scale compared to A1 to highlight the slight inhomogeneity) and final total Dsh* distribution for a fixed feedback strength and different diffusion strength. The remaining parameter values are presented in Table S7. Columns 2 and 3: For these parameter values vertex polarity is not robust to noise in the initial Ld distribution. Column 4: For sufficiently strong diffusion both initial conditions yield the unpolarised steady state. (TIF) [file pone.0060064.s004.tif]

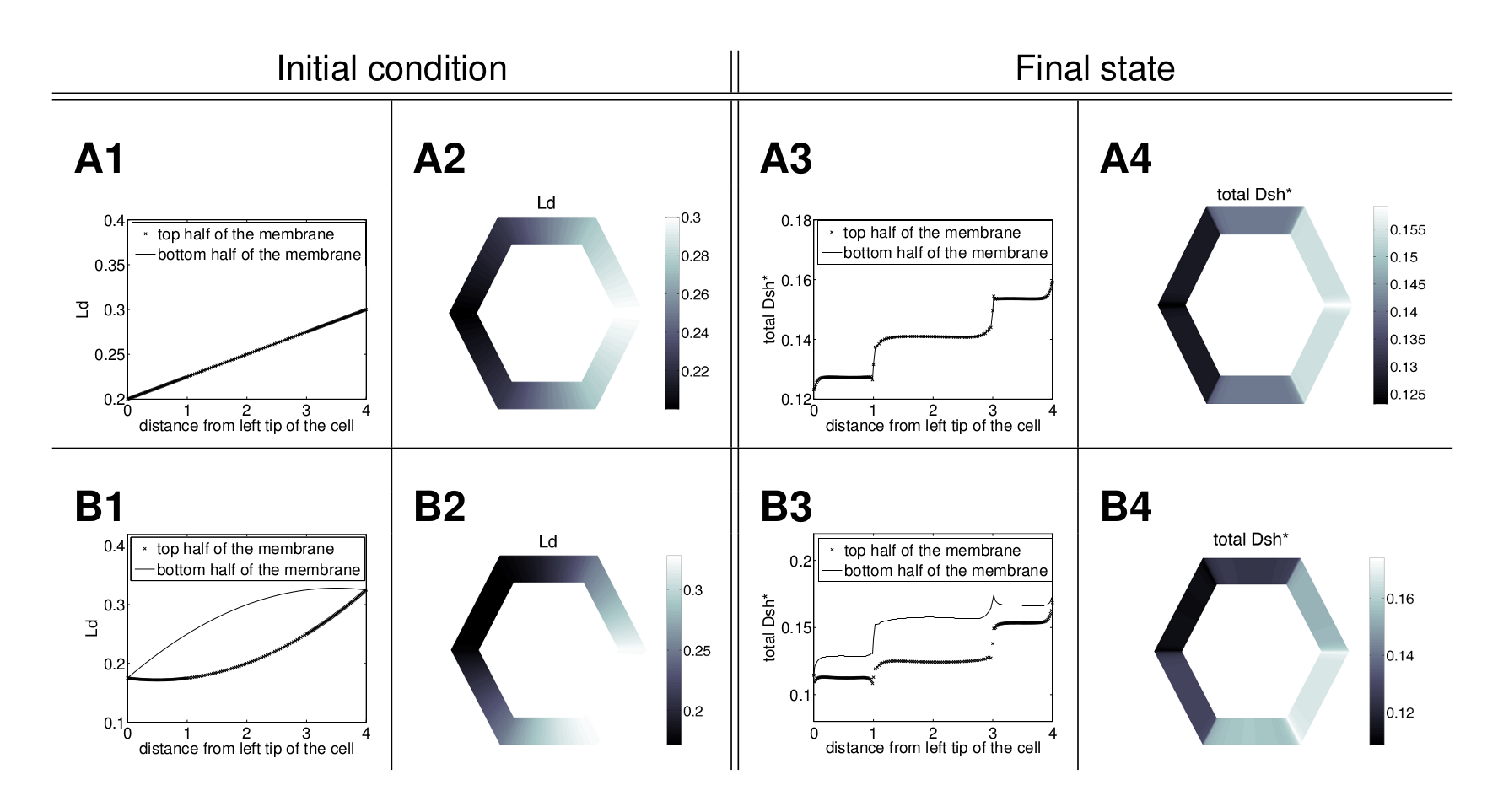

Supplement: Figure S5 — Final distribution of total Dsh* for the full spatial version of Model L for different initial conditions. Protein and protein complex distributions occur on the membrane. In every case a line plot and a two-dimensional plot are shown. The corresponding parameter values are shown in Table S8. Row A: an initial ligand distribution that is weakly vertex polarised yields vertex polarity of total Dsh*. However, this state is not stable to perturbations that break the anterior-posterior symmetry. Row B: an initially side polarised ligand distribution yields a side polarised distribution of total Dsh*. The line plots show top and bottom half of the cell separately. (TIF) [file pone.0060064.s005.tif]
