# Supplementary material for: Is a Persistent Global Bias Necessary for the Establishment of Planar Cell Polarity?
Source: PLoS One. 2013 Apr 8;8(4):e60064. doi: 10.1371/journal.pone.0060064 (PMC3620226; doi:10.1371/journal.pone.0060064)
Supplement: Table S1 — Set of parameter values for which Model A, exemplified by equation (S1), can generate polarity with and without the global bias. We used these parameter values for the simulations shown in Figures 4, 6 and 8 in the main text. (PDF) [file pone.0060064.s006.pdf]

K_b	10
K_p	2.2
K_{pk}	0.5
K_{va}	0.5
μ	$(0.1, 0.1, 0.1, 0.1, 0.001, 0.1)$
λ	$(0.1, 0.1, 0.1, 0.1, 0.01, 0.1, 0.1, 0.1, 0.1, 0.1)$
R	$(10, 5, 5, 5, 10, 5, 5, 10, 5, 5)$
