# Supplementary material for: Is a Persistent Global Bias Necessary for the Establishment of Planar Cell Polarity?
Source: PLoS One. 2013 Apr 8;8(4):e60064. doi: 10.1371/journal.pone.0060064 (PMC3620226; doi:10.1371/journal.pone.0060064)
Supplement: Table S2 — Choice of parameter values which were used for the parameter scan for the two dimensional, compartmentalised version of Model A in the main text. The values of the remaining parameters are the same as in Table S1. The diffusion depends on a parameter that was varied to gain insight into the effect of the speed of diffusion on the final state. The parameter is varied to investigate the influence of the feedback strength on the steady states. These parameter values were used to obtain Figures 10 and 11 in the main text. (PDF) [file pone.0060064.s007.pdf]

K_b	20
K_p	K_p
μ	$m \cdot (0.1, 0.1, 0.1, 0.1, 0.001, 0.1)$
