# Supplementary material for: Is a Persistent Global Bias Necessary for the Establishment of Planar Cell Polarity?
Source: PLoS One. 2013 Apr 8;8(4):e60064. doi: 10.1371/journal.pone.0060064 (PMC3620226; doi:10.1371/journal.pone.0060064)
Supplement: Table S3 — Set of parameter values for simulations of Model A in Figure S1. (PDF) [file pone.0060064.s008.pdf]

K_{pk}	0.5
K_{va}	0.5
K_b	500
K_p	5
μ	$(100, 100, 0.1, 0.1, 0.01, 0.1, 0.1, 0.1, 0.1, 0.1)$
λ	$(50, 25, 0.001, 25, 50, 25, 25, 50, 25, 25)$
R	$(100, 50, 1000, 50, 100, 50, 50, 100, 50, 50)$
