# Supplementary material for: Is a Persistent Global Bias Necessary for the Establishment of Planar Cell Polarity?
Source: PLoS One. 2013 Apr 8;8(4):e60064. doi: 10.1371/journal.pone.0060064 (PMC3620226; doi:10.1371/journal.pone.0060064)
Supplement: Table S4 — Set of parameter values from [8] after rescaling of the diffusion coefficients. We used these values for the simulations of Model L in Figures S2 and S3 as well as in Figure 5 in the main text. (PDF) [file pone.0060064.s009.pdf]

A_3	1.5
A_5	1.5
A_8	1.5
B_3	3
B_5	3
B_8	3
Kf	$(0.01, 0.01, 0.01, 0.015, 0.03, 0.03, 0.003, 0.003)$
Kd	$(0, 0.04, 0.04, 0.04, 0.001, 0.001, 0.0005, 0.0005)$
μ	$1.27(0.02, 0.02, 0.02, 0.02, 0.02, 0.02, 0.02, 0.02, 0.02)$
