# Supplementary material for: Is a Persistent Global Bias Necessary for the Establishment of Planar Cell Polarity?
Source: PLoS One. 2013 Apr 8;8(4):e60064. doi: 10.1371/journal.pone.0060064 (PMC3620226; doi:10.1371/journal.pone.0060064)
Supplement: Table S5 — Parameter values for which Model L can have a polarising instability in the absence of a ligand gradient. Entry represents the maximal real part of the eigenvalues of the system exemplified by equation (S5). The corresponding eigenvector is given in Table S6. These parameter values were used to generate Figures 7 and 8 in the main text. (PDF) [file pone.0060064.s010.pdf]

A_3	3.3885
A_5	8.5385
A_8	0.8
B_3	6.0579
B_5	10.8
B_8	0.5385
Kf	$(10.3185, 5.5385, 6.0385, 2.3535,$ $13.5095, 1.8358, 10.5385, 11.3882)$
Kd	$(0, 0.9965, 1.1021, 1.4912,$ $0.6353, 0.4319, 2.7598, 0.4319)$
μ	$(0.0625, 0.0625, 0.0019, 0.0625,$ $0.1250, 0.0625, 0.0625, 0.1250, 0.0019)$
λ	0.0081
