# Supplementary material for: Is a Persistent Global Bias Necessary for the Establishment of Planar Cell Polarity?
Source: PLoS One. 2013 Apr 8;8(4):e60064. doi: 10.1371/journal.pone.0060064 (PMC3620226; doi:10.1371/journal.pone.0060064)
Supplement: Table S6 — For Model L the parameter values in Table S5 can give polarised distributions of the protein complexes. Eigenvector corresponding to the eigenvalue for the homogeneous unpolarised steady state of the system exemplified by equation (S5) and the parameter values in Table S5. To reduce the computational effort we applied the conservation laws for the six proteins and therefore there are no entries corresponding to and . (PDF) [file pone.0060064.s011.pdf]

	left	right
Ld	–	0
Fz	–	0
Fz*	-0.0065	0.0065
Fmi	–	-0.0575
Vang	–	-0.299
Dsh	–	0.0444
Pk	–	-0.0509

	left	right
Fz*Fmi	-0.0277	0.0277
FmiVang	-0.0433	0.0433
Fz*FmiFmiVang	0.0116	-0.0116
Dsh*FzFmiFmiVang	0.0430	-0.0430
Fz*FmiFmiVaPk	0.1496	-0.1496
Dsh*FzFmiFmiVaPk	0.6845	-0.6845
