# Supplementary material for: Is a Persistent Global Bias Necessary for the Establishment of Planar Cell Polarity?
Source: PLoS One. 2013 Apr 8;8(4):e60064. doi: 10.1371/journal.pone.0060064 (PMC3620226; doi:10.1371/journal.pone.0060064)
Supplement: Table S7 — Choice of parameter values for Model L with variable feedback and diffusion strength. The parameter values for the diffusion coefficients from Table S5 are multiplied by a parameter . This was varied in the parameter scan for the two dimensional compartmentalised version of Model L in the main text to gain insight into the effect of the speed of diffusion on the final state. The feedback parameters and from Table S5 were all multiplied by the same parameter and was varied to investigate the influence of the feedback on the final state. The parameter values for and are the same as in Table S5. This parameter set was used to generate Figure S4 as well as Figure 11 in the main text. (PDF) [file pone.0060064.s012.pdf]

A_3	$3.3885F$
A_5	$8.5385F$
A_8	$0.8F$
B_3	$6.0579F$
B_5	$10.8F$
B_8	$0.5385F$
μ	$D \cdot (0.0625, 0.0625, 0.0019, 0.0625, 0.1250, 0.0625, 0.0625, 0.1250, 0.0019)$
