# Supplementary material for: Is a Persistent Global Bias Necessary for the Establishment of Planar Cell Polarity?
Source: PLoS One. 2013 Apr 8;8(4):e60064. doi: 10.1371/journal.pone.0060064 (PMC3620226; doi:10.1371/journal.pone.0060064)
Supplement: Table S8 — Parameter values for the simulations of Model L, exemplified by (S7), in Figure S5. (PDF) [file pone.0060064.s013.pdf]

A_3	2.9577
A_5	8.1077
A_8	0.3692
B_3	5.6271
B_5	10.3692
B_8	0.1077
Kf	$(858.8877, 398.1077, 86.6077, 57.9227, 12.3716, 1.405, 10.1077, 10.9574)$
Kd	$(0, 0.5657, 0.6713, 0.3533, 0.2045, 0.001114, 0.9148, 0.001177)$
μ	$4 \cdot (0.7519, 0.4829, 0.1695, 0.7507, 2.6369, 1.4969, 1.5244, 2.4982, 0.0269)$
