# Supplementary material for: Is a Persistent Global Bias Necessary for the Establishment of Planar Cell Polarity?
Source: PLoS One. 2013 Apr 8;8(4):e60064. doi: 10.1371/journal.pone.0060064 (PMC3620226; doi:10.1371/journal.pone.0060064)
Supplement: Supporting Information S1 — Example equations for the different discretisations of Models A and L as well as parts of the analysis that are beyond the scope of the main text. (PDF) [file pone.0060064.s014.pdf]

Supporting Information S1: Is a persistent global bias necessary for the establishment of planar cell polarity?

Sabine Fischer, Paul Houston, Nicholas A. M. Monk, Markus R. Owen

A Model A (adapted from Amonlirdviman *et al.* [1])

A.1 One spatial dimension

We assume a row of ten two-sided cells. On each side of a cell there are certain concentrations of the four proteins Dsh, Fz, Vang and Pk. At the two ends of the row we apply periodic boundary conditions. Intracellular diffusion takes place as exchange between the two sides of a cell of all proteins and protein complexes that are not cell-bridging. Applying the law of mass action to reactions (1)–(10) in the main text yields a system of ODEs for cell i, which describes the protein interactions taking place. Here, we present two sample equations:

$$
\begin{aligned}
\frac{\mathrm{d}\,[Dsh]_i^l}{\mathrm{d}\,t} &= -R_1\,[Dsh]_i^l\,[Fz]_i^l + \lambda_1 B_i^l\,[DshFz]_i^l - R_5\,[Dsh]_i^l\,[FzVang]_{i-1}^r \\
&\quad + \lambda_5 B_i^l\,[DshFzVang]_{i-1}^r - R_8\,[Dsh]_i^l\,[FzvVangPk]_{i-1}^r \\
&\quad + \lambda_8 B_i^l\,[DshFzVangPk]_{i-1}^r + \mu_1([Dsh]_i^r - [Dsh]_i^l),
\end{aligned}
\tag{S1}
$$

$$
\begin{aligned}
\frac{\mathrm{d}\,[Dsh]_i^r}{\mathrm{d}\,t} &= -R_1\,[Dsh]_i^r\,[Fz]_i^r + M_1\lambda_1 B_i^r\,[DshFz]_i^r - R_5\,[Dsh]_i^r\,[FzVang]_{i+1}^l \\
&\quad + M_1\lambda_5 B_i^r\,[DshFzVang]_{i+1}^l - R_8\,[Dsh]_i^r\,[FzVangPk]_{i+1}^l \\
&\quad + M_1\lambda_8 B_i^r\,[DshFzVangPk]_{i+1}^l + \mu_1([Dsh]_i^l - [Dsh]_i^r),
\end{aligned}
$$

with

$$
\begin{aligned}
B_i^l = 1 + K_b(&K_{Pk}\,[Pk]_i^l + [VangPk]_i^l + [FzVangPk]_i^l + [DshFzVangPk]_i^l \\
&+ K_{va}([Vang]_i^l + [FzVang]_i^l + [DshFzVang]_i^l))^{K_p}
\end{aligned}
$$

$$
\begin{aligned}
B_i^r = 1 + K_b(&K_{pk}\,[Pk]_i^r + [VangPk]_i^r + [FzVangPk]_i^r + [DshFzVangPk]_i^r \\
&+ K_{va}([Vang]_i^r + [FzVang]_i^r + [DshFzVang]_i^r))^{K_p}
\end{aligned}
$$

and

$$M_1 < 1.$$

The superscripts ^{l} and ^{r} refer to the cell sides, left and right, respectively, while the subscripts refer to the number of the cell. The square brackets indicate that we are dealing with concentrations. Bridging complexes are always counted as if they belong to the cell in which their Vang-part is located. Diffusion is introduced by the parameter $\mu_1 = \frac{\tilde{\mu}_1}{\Delta x^2}$ with the diffusion coefficient $\tilde{\mu}_1$ and the spatial extension of the model cell from left to right $\Delta x = 8\mu m$ [2].
As mentioned above and indicated by (S1) the proposed mechanism relies on intracellular protein

movement. Protein movement between cells and production or degradation of proteins are not considered, i.e. the model is conservative. There are four conservation laws, one for each protein. We found that using these laws to reformulate the equations does not give us more insight into the model and therefore we omit it.
The parameter values chosen by Amonlirdviman *et al.* for their simulations in [1] vary in orders of magnitude between 10^{-5} and 10^5. Such a set of parameter values would considerably slow down our simulations. Furthermore, these values are not based on experimental estimates. Therefore, we chose different parameter values as shown in Table S1. However, Amonlirdviman *et al.* conducted a sensitivity analysis which gave them a range for each parameter value in which it could vary such that the model still yields wild-type polarity. Except for μ_5 and λ_5 all our parameter values lie within the respective ranges.

A.2 Two spatial dimensions for compartmentalised cells

In the second discretisation, we consider one hexagonal cell in two spatial dimensions, which is divided into six compartments. To represent a whole field of cells with identical initial conditions, we apply periodic boundary conditions for the intercellular interactions. Within the cell, diffusion occurs between a given compartment and its two neighbours for all proteins and protein complexes that do not bridge cells. Applying the law of mass action to the reactions (1)–(10) in the main text and omitting the persistent global bias, we get a system of ODEs describing the rate of change of a protein or protein complex concentration in compartment j of cell i. As an example, we present the equation for the change of $[Dsh]$ in compartment j (mod 6) of cell i represented by $[Dsh]_{i,j}$. It is

$$\begin{aligned}\frac{\mathrm{d}\,[Dsh]_{i,j}}{\mathrm{d}\,t} = &-R_1\,[Dsh]_{i,j}\,[Fz]_{i,j} + \lambda_1 B_{i,j}\,[DshFz]_{i,j} - R_5\,[Dsh]_{i,j}\,[FzVang]^+_{i,j}\\ &+ \lambda_5 B_{i,j}\,[DshFzVang]^+_{i,j} - R_8\,[Dsh]_{i,j}\,[FzVangPk]^+_{i,j}\\ &+ \lambda_8 B_{i,j}\,[DshFzVangPk]^+_{i,j} + \mu_1([Dsh]_{i,j+1} + [Dsh]_{i,j-1} - 2[Dsh]_{i,j}),\end{aligned} \tag{S2}$$

with

$$\begin{aligned}B_{i,j} = 1 + K_b(&K_{pk}[Pk]_{i,j} + [VangPk]_{i,j} + [FzVangPk]_{i,j} + [DshFzVangPk]_{i,j}\\ &+ K_{va}([Vang]_{i,j} + [FzVang]_{i,j} + [DshFzVang]_{i,j}))^{K_p},\end{aligned}$$

where $^+$ indicates that the reactants are in adjacent compartments of neighbouring cells. The parameter $\mu_1 = \frac{\tilde{\mu}_1}{\Delta x^2}$, where $\tilde{\mu}_1$ is the diffusion coefficient and Δx is the distance between two neighbouring compartments within a cell. We assume $\Delta x = 2\mu m$.
To obtain the steady states of the system exemplified by (S2) for different parameter values, we conduct a parameter scan as described in the main text. The parameter values are shown in Table S2 and we vary K_p and m to investigate the impact of the feedback strength and the diffusion on the stability of the different polarised steady states.

A.3 Full spatial model

Here, we consider the full spatial model on one hexagonal domain. Instead of compartmentalising the cell as in Section A.2 we now approximate the full system of partial differential equations for diffusion within the cell and the membrane. Applying reaction kinetics to the reactions (1)–(10) in the main text and omitting the persistent global bias, we obtain the desired system of partial differential equations. Here, we present the equation for $[Dsh]$ as an example. To this end, we have

$$\begin{aligned}\frac{\partial\,[Dsh]}{\partial t} = &-R_1\,[Dsh][Fz] + \lambda_1 B\,[DshFz] - R_5\,[Dsh][FzVang]^+ + \lambda_5 B\,[DshFzVang]^+\\ &- R_8\,[Dsh][FzVangPk]^+ + \lambda_8 B\,[DshFzVangPk]^+ + \mu_1\,\nabla^2[Dsh]\end{aligned}, \tag{S3}$$

where

$$B = 1 + K_b(K_{pk}[Pk] + [VangPk] + [FzVangPk] + [DshFzVangPk] + K_{va}([Vang] + [FzVang] + [DshFzVang]))^{K_p}. \tag{S4}$$

The superscript $^+$ indicates that the reactants are in different cells and μ_1 is the diffusion coefficient. We assume that the side length of a cell is $2\mu m$.

We simulated the system exemplified by (S3) in Matlab applying the finite element method [3]. We assumed a hexagonal domain and periodic boundary conditions for the intercellular interactions to represent a whole field of cells with identical initial conditions. The different partial differential equations underlying the model are numerically approximated on different parts of the hexagonal domain, depending on where the corresponding proteins and protein complexes occur in the biological cell. In this respect, the ten proteins and protein complexes can be divided into three groups. Dsh and Pk have been observed in the cytoplasm, i.e. in the whole hexagonal domain in our simulations; they are freely diffusible in this region. Therefore, we simulate the partial differential equations corresponding to these proteins on the whole of the two-dimensional hexagonal domain, applying Neumann boundary conditions which are determined by the reaction equations of interactions of Dsh and Pk with membrane located proteins. The components Fz, Vang, DshFz and VangPk are found in the cell membrane, diffusing within these constraints. The corresponding partial differential equations for these quantities are therefore simulated on a one-dimensional domain with periodic boundary conditions. The cell bridging complexes FzVang, DshFzVang, FzVangPk and DshFzVangPk occupy only the part of the membrane which is common to the two cells they connect. They can diffuse in this part but cannot move past a vertex of the biological cell. Therefore, the corresponding partial differential equations for these complexes are simulated on six one-dimensional domains, one for every edge of the biological cell, together with homogeneous Neumann boundary conditions.
Figure S1 shows two initial conditions and the corresponding final states for the sum of the Dsh complexes. All the proteins and protein complexes shown in this figure only occur on the membrane. For clarity, we show a line plot and a two-dimensional representation in each case.

The initial condition in row A of Figure S1 has a weak initial vertex polarity whereas the initial condition in row B is weakly polarised to the side. In contrast to the previous sections we found that an initial imbalance in Fz could not yield significant polarity (not shown). Figure S1 row A shows that a weak initial vertex polarity in the Vang concentration yields vertex polarity. Figures S1 B1 and B2 show a weak initial side polarity in Vang. The other proteins are initially distributed homogeneously. The final state in B3 and B4 shows that the side polarity remains throughout the simulations.
If we increase the diffusion in Figure S1, the imbalance between the different parts of the cell becomes weaker and for a sufficiently large diffusion we get the unpolarised steady state.
The results show that vertex polarity is not stable to asymmetric perturbations which is consistent with the findings in the main text.

B Model L (adapted from Le Garrec *et al.* [4])

B.1 One spatial dimension

In the one-dimensional discretisation of Model L, we consider a row of two-sided cells. On each side of a cell there are certain amounts of the proteins and protein complexes. All proteins and protein complexes that do not bridge between neighbouring cells are free to diffuse, i.e. change position from one side of the cell to the other. Applying the law of mass action to reactions (11)–(18) in the main text yields a system of ODEs for the rates of change of the protein and protein complex concentrations

in cell i. We present two sample equations:

$$\begin{aligned}\frac{\mathrm{d}\,[Pk]_i^l}{\mathrm{d}\,t} &= -inh5_i^l Kf_5\,[Fz^*FmiFmiVang]_{i-1}^r \cdot [Pk]_i^l + en5_i^l Kd_5\,[Fz^*FmiFmiVangPk]_{i-1}^r \\ &\quad - inh8_i^l Kf_8\,[Dsh^*FzFmiFmiVang]_{i-1}^r \cdot [Pk]_i^l \\ &\quad + en8_i^l Kd_8\,[Dsh^*FzFmiFmiVangPk]_{i-1}^r \\ &\quad + \mu_7\,([Pk]_i^r - [Pk]_i^l), \\ \frac{\mathrm{d}\,[Pk]_i^r}{\mathrm{d}\,t} &= -inh5_i^r Kf_5\,[Fz^*FmiFmiVang]_{i+1}^l \cdot [Pk]_i^r + en5_i^r Kd_5\,[Fz^*FmiFmiVangPk]_{i+1}^l \\ &\quad - inh8_i^r Kf_8\,[Dsh^*FzFmiFmiVang]_{i+1}^l \cdot [Pk]_i^r \\ &\quad + en8_i^r Kd_8\,[Dsh^*FzFmiFmiVangPk]_{i+1}^l \\ &\quad + \mu_7\,([Pk]_i^l - [Pk]_i^r),\end{aligned} \tag{S5}$$

where

$$inh5_i^l = \frac{1}{1 + A_5([Dsh^*FzFmiFmiVang]_i^l + [Dsh^*FzFmiFmiVangPk]_i^l)},$$

$$inh5_i^r = \frac{1}{1 + A_5([Dsh^*FzFmiFmiVang]_i^r + [Dsh^*FzFmiFmiVangPk]_i^r)},$$

$$en5_i^l = 1 + B_5([Dsh^*FzFmiFmiVang]_i^l + [Dsh^*FzFmiFmiVangPk]_i^l),$$

$$en5_i^r = 1 + B_5([Dsh^*FzFmiFmiVang]_i^r + [Dsh^*FzFmiFmiVangPk]_i^r),$$

and $inh8$ and $en8$ are defined analogously. The parameter $\mu_7 = \frac{\tilde{\mu}_7}{\Delta x^2}$, where $\tilde{\mu}_7$ is the diffusion coefficient and $\Delta x = 8\mu m$ the spatial extension of a cell from left to right [2]. The equations describe the variation of the protein concentrations, indicated by the square brackets. Subscripts refer to the cell number, superscripts to the cell side, left or right. Bridging complexes do not diffuse and they are counted as if they belong to the same cell as their Fz-part. The system exemplified by (S5) conserves the total concentration of each protein in a cell. However, reformulating the equations using the conservation laws would not simplify the system significantly.

To use the parameter values chosen in [4] for our setting, we require the appropriate scaling for the diffusion coefficient. In [4] they consider 150 roughly hexagonal cells on a screen of 175 by 175 pixels, whereby each pixel has a size of 1 µm. Hence, the side length Δx^{hex} of one cell is about 9 µm. Let μ_L denote the dimensionless diffusion coefficient for the hexagonal cells in [4] and μ the dimensionless diffusion coefficient for our one-dimensional analysis. With $\mu_L = \frac{\tilde{\mu}_L}{81\mu m}$ we then get

$$\mu = \frac{\tilde{\mu}_L}{64\mu m} = \mu_L \frac{81\mu m}{64\mu m} = 1.27\mu_L.$$

The parameter values are shown in Table S4.

Initial ligand gradient To facilitate the comparison of the results of the two models, we used the same deterministic approach as before to analyse Model L, although Le Garrec *et al.* used a stochastic approach in their work [4]. To ensure that our modified version of the model gives similar results to the original one we commenced our analysis by simulating the model in Matlab for the initial ligand gradient and the parameter values given in [4], adapted to the geometry in our simulations.

We chose the concentrations of the proteins in each membrane pixel in [4] as our initial protein concentrations. Hence, initially Fz, Fmi, Vang, Dsh and Pk are distributed homogeneously in every cell, with $[Fz]_i^l = [Fz]_i^r = 4, [Fmi]_i^l = [Fmi]_i^r = 4, [Vang]_i^l = [Vang]_i^r = 2, [Dsh]_i^l = [Dsh]_i^r = 2$ and $[Pk]_i^l = [Pk]_i^r = 2$ for all cells i. As boundary conditions we assumed that at both ends of the row we have boundary cells (cells 1 and N) in which $c_1^l = 0, c_1^r = c_2^l, c_N^l = c_{N-1}^r$ and $c_N^r = 0$, where c_i^l and c_i^r are the concentrations of any protein or protein complex in cell i on the left and right side, respectively. Furthermore, all intracellular diffusion coefficients in cell 1 and cell N are zero; the remaining interactions in these cells are governed by the same equations as for the rest of the cells. In [4] there are roughly 13 cells in each row. Therefore, we simulated the system for 11 cells plus 2 boundary cells. Figure S2 shows the corresponding results. We present the final distribution of the sum of the Dsh* complexes, since in [4] this is assumed to determine the direction of the hair growth; the hairs are assumed to grow at the end of the cell with the highest Dsh* concentration. We see that our modified version of the model gives similar results to the original model in [4], in that we obtain polarity to the right in every cell. Due to the boundary conditions, the polarity of the cells at the two ends of the row is weaker than that of cells in the middle of the row.

Weak initial ligand imbalance in every cell In the second part of the one-dimensional analysis of Model L we consider a row of ten cells with periodic boundary conditions and an initial weak ligand imbalance in every cell. Figure S3 shows that for the parameter values in Table S4, even for a strong initial cue the system exemplified by equation (S5) cannot generate polarity. This is supported by linear stability analysis; we calculated the homogeneous unpolarised steady state for the given total amounts of the proteins and the parameter set in Table S4. Subsequently, we evaluated the eigenvalue with the greatest real part λ. In this case it is $\lambda = -1.4 \cdot 10^{-4} < 0$, i.e. the unpolarised steady state is stable to any small perturbation. Hence, linear stability analysis does not predict instabilities that can yield polarisation for this set of parameter values.

The aim then was to find a parameter set for which the system polarises for a small initial ligand imbalance in every cell. To this end, we applied the Nelder-Mead method to identify a set of parameter values, for which the unpolarised steady is unstable and the eigenvalue with the largest real part λ corresponds to an eigenvector which is associated with a polarised state. The value of the objective function f for a given parameter set x_i was obtained by calculating the corresponding homogeneous unpolarised steady state and the eigenvalue with the largest real part λ_i. Then $f(x_i) = -\lambda_i$ and f was minimised. The resulting parameter set and its corresponding λ are shown in Table S5, while the eigenvector associated to λ is presented in Table S6. Figures 7 and 8 in the main text show that indeed for the parameter values in Table S5 the system exemplified by (S5) can generate polarity from a weak initial ligand imbalance in every cell.

B.2 Two spatial dimensions for compartmentalised cells

In this discretisation, we assume one hexagonal cell which contains six compartments. Proteins and protein complexes that do not bridge the membrane can diffuse between neighbouring compartments. By applying periodic boundary conditions our setup represents a whole field of cells with identical initial conditions in every cell. To obtain the relevant system of equations we apply reaction kinetics to the reactions (11)-(18) in the main text. We present one sample equation.

$$
\begin{aligned}
\frac{\mathrm{d}\,[Pk]_{i,j}}{\mathrm{d}\,t} = &-inh5_{i,j}Kf_5\;[Fz^*FmiFmiVang]^+_{i,j}\cdot[Pk]_{i,j}\\
&+en5_{i,j}Kd_5\;[Fz^*FmiFmiVangPk]^+_{i,j}\\
&-inh8_{i,j}Kf_8\;[Dsh^*FzFmiFmiVang]^+_{i,j}\cdot[Pk]_{i,j}\\
&+en8_{i,j}Kd_8\;[Dsh^*FzFmiFmiVangPk]^+_{i,j}\\
&+\mu_7[Pk]_{i,j+1}+[Pk]_{i,j-1}-2[Pk]_{i,j},
\end{aligned}
\tag{S6}
$$

where

$$
\begin{aligned}
inh5_{i,j} &= \frac{1}{1+A_5([Dsh^*FzFmiFmiVang]_{i,j}+[Dsh^*FzFmiFmiVangPk]_{i,j})},\\
en5_{i,j} &= 1+B_5([Dsh^*FzFmiFmiVang]_{i,j}+[Dsh^*FzFmiFmiVangPk]_{i,j})
\end{aligned}
$$

and $inh8$ and $en8$ are defined analogously. The superscript $^+$ indicates binding over the cell membrane, i is the cell number and j (mod 6) the number of the compartment. The diffusion is introduced by $\mu_7 = \frac{\tilde{\mu_7}}{\Delta x}$, where $\tilde{\mu_7}$ is the diffusion coefficient and $\Delta x = 2\mu m$ is the distance between two neighbouring compartments within a cell.
For this setting we conduct a parameter scan to identify the stable steady states as described in the main text. Thereby we focus on the effect of the feedback and diffusion strength on the steady state of the system. We use the parameter values in Table S7, in which varying F changes the feedback strength and varying D the diffusion strength. Figure S4 shows examples of steady states for a fixed feedback strength and varying diffusion coefficients. We see that for a weak initial vertex polarity in Ld and sufficiently weak diffusion the final Dsh* distribution is vertex polarised. However, for the same parameter values and an inhomogeneous initial condition the model yields the triangular state or side polarity, indicating that vertex polarity is unstable for these parameter values. For both initial conditions, increasing the diffusion yields the homogeneous unpolarised steady state.

B.3 Full spatial model

In this section we consider a full spatial version of Model L on a hexagonal domain. Rather than a coarse compartmentalisation of the cells as in Section B.2, we simulate the full partial differential model for reaction and diffusion within the cell membrane. In [4] Le Garrec *et al.* assume that the cell bridging complexes cannot diffuse. Furthermore, they found that in their model cytoplasmic diffusion does not significantly alter the polarisation. Therefore, we assume that all proteins and complexes occur in the membrane and diffuse within this domain unless they are bridging complexes. We obtain the system of equations by applying the law of mass action to the reactions (11)–(18) in the main text. Here, we present the equation for $[Pk]$ as an example. To this end, we get

$$
\begin{aligned}
\frac{\partial\,[Pk]}{\partial t} = &-inh5\,Kf_5\;[Fz^*FmiFmiVang]^+\;[Pk] + en5\,Kd_5\;[Fz^*FmiFmiVangPk]^+\\
&-inh8\,Kf_8\;[Dsh^*FzFmiFmiVang]^+\;[Pk]\\
&+en8\,Kd_8\;[Dsh^*FzFmiFmiVangPk]^+ + \mu_7\nabla^2[Pk],
\end{aligned}
\tag{S7}
$$

where

$$
\begin{aligned}
inh5 &= \frac{1}{1+A_5([Dsh^*FzFmiFmiVang]+[Dsh^*FzFmiFmiVangPk])},\\
inh8 &= \frac{1}{1+A_8([Dsh^*FzFmiFmiVang]+[Dsh^*FzFmiFmiVangPk])},
\end{aligned}
$$

$$en5 = 1 + B_5([Dsh^* FzFmiFmiVang] + [Dsh^* FzFmiFmiVangPk]),$$
$$en8 = 1 + B_8([Dsh^* FzFmiFmiVang] + [Dsh^* FzFmiFmiVangPk]).$$

The superscript $^+$ indicates binding over the cell membrane and μ_7 is the diffusion coefficient. The side length of our hexagonal domain is $2\mu m$. Our choice of parameter values is presented in Table S8. This parameter set was also obtained by the Nelder-Mead optimisation of the eigenvalue with the largest real part described in Section B.1.

We numerically approximated the system exemplified by (S7) in Matlab, applying the finite element method. Since in this model all proteins and protein complexes that diffuse do so in the whole membrane, our problem is essentially one-dimensional with periodic boundary conditions [3].
We consider one hexagonal cell and apply periodic boundary conditions for the cell bridging complex formation. Therefore, our setup represents a whole field of cells with identical initial conditions and dynamics.
Figure S5 shows different initial conditions and the corresponding final distributions of total Dsh*. In each case we show a line plot and a two-dimensional representation. In Figure S5 row A, both the initial condition and the final state show a vertex polarity. We see that an initial ligand imbalance in the cell can lead to polarisation of the Dsh* distribution. Figure S5 row B shows that an initial ligand distribution that is weakly side polarised yields a side polarised distribution of total Dsh*. The line plots show the distributions on the top and bottom half of the cell separately. Increasing the diffusion in row A and B weakens the difference of total Dsh* between the different parts of the cell.
These results are consistent with our findings in the main text; the vertex polarised state is unstable to asymmetric perturbations.

References

[1] Amonlirdviman K, Khare NA, Tree DRP, Chen WS, Axelrod JD, et al. (2005) Mathematical modeling of planar cell polarity to understand domineering nonautonomy. Science 307: 423-426.

[2] Kacmarczyk T, Craddock EM (2000) Cell size is a factor in body size variation among Hawaiian and nonHawaiian species of *Drosophila*. Dros Inf Serv 83: 144-148.

[3] Schamberg S (2009) Modelling planar cell polarity in *Drosophila melanogaster*. Ph.D. thesis, School of Mathematical Sciences, University of Nottingham, UK.

[4] Le Garrec JF, Lopez P, Kerszberg M (2006) Establishment and maintenance of planar epithelial cell polarity by asymmetric cadherin bridges: A computer model. Dev Dyn 235: 235-246.
